# Functional connectivity predictors and mechanisms of symptom change in functional neurological disorder

**DOI:** 10.1093/braincomms/fcag290

**Published:** 2026-07-22

**Authors:** Christiana Westlin, Cristina Bleier, Andrew J Guthrie, Sara A Finkelstein, Julie Maggio, Jessica Ranford, Julie MacLean, Ellen Godena, Daniel Millstein, Jennifer Freeburn, Caitlin Adams, Christopher D Stephen, Ibai Diez, David L Perez

**Affiliations:** Mass General Brigham Department of Neurology, Massachusetts General Hospital, Harvard Medical School, Boston, MA 02114, USA; Mass General Brigham Department of Psychiatry, Massachusetts General Hospital, Harvard Medical School, Boston, MA 02114, USA; Athinoula A. Martinos Center for Biomedical Imaging, Massachusetts General Hospital, Harvard Medical School, Boston, MA 02129, USA; Mass General Brigham Department of Neurology, Massachusetts General Hospital, Harvard Medical School, Boston, MA 02114, USA; Athinoula A. Martinos Center for Biomedical Imaging, Massachusetts General Hospital, Harvard Medical School, Boston, MA 02129, USA; Mass General Brigham Department of Neurology, Massachusetts General Hospital, Harvard Medical School, Boston, MA 02114, USA; Athinoula A. Martinos Center for Biomedical Imaging, Massachusetts General Hospital, Harvard Medical School, Boston, MA 02129, USA; Mass General Brigham Department of Neurology, Massachusetts General Hospital, Harvard Medical School, Boston, MA 02114, USA; Department of Physical Therapy, Massachusetts General Hospital, Boston, MA 02114, USA; Department of Occupational Therapy, Massachusetts General Hospital, Boston, MA 02114, USA; Department of Occupational Therapy, Massachusetts General Hospital, Boston, MA 02114, USA; Mass General Brigham Department of Neurology, Massachusetts General Hospital, Harvard Medical School, Boston, MA 02114, USA; Mass General Brigham Department of Neurology, Massachusetts General Hospital, Harvard Medical School, Boston, MA 02114, USA; Mass General Brigham Department of Psychiatry, Massachusetts General Hospital, Harvard Medical School, Boston, MA 02114, USA; Department of Speech, Language, and Swallowing Disorders, Massachusetts General Hospital, Harvard Medical School, Boston, MA 02114, USA; Mass General Brigham Department of Neurology, Massachusetts General Hospital, Harvard Medical School, Boston, MA 02114, USA; Mass General Brigham Department of Psychiatry, Massachusetts General Hospital, Harvard Medical School, Boston, MA 02114, USA; Mass General Brigham Department of Neurology, Massachusetts General Hospital, Harvard Medical School, Boston, MA 02114, USA; Computational Neuroimaging Lab, Biobizkaia Health Research Institute, Barakaldo 48903, Spain; Ikerbasque Basque Foundation for Science, Bilbao 48903, Spain; Center for Inflammation Imaging, Department of Radiology, Massachusetts General Hospital, Harvard Medical School, Boston, MA 20114, USA; Mass General Brigham Department of Neurology, Massachusetts General Hospital, Harvard Medical School, Boston, MA 02114, USA; Mass General Brigham Department of Psychiatry, Massachusetts General Hospital, Harvard Medical School, Boston, MA 02114, USA; Athinoula A. Martinos Center for Biomedical Imaging, Massachusetts General Hospital, Harvard Medical School, Boston, MA 02129, USA; Mass General Brigham Departments of Neurology and Psychiatry, Brigham and Women’s Hospital, Harvard Medical School, Boston, MA 20115, USA

**Keywords:** functional movement disorder, functional seizures, fMRI, graph theory, outcomes

## Abstract

Clinical trajectories in patients with functional neurological disorder (FND) are variable, and the neural mechanisms underlying this heterogeneity remain poorly understood. This longitudinal brain imaging study examined resting-state functional connectivity predictors and mechanisms of symptom change in FND. Thirty-two adults with FND (motor and/or seizure phenotypes) completed baseline questionnaires and functional MRI (fMRI), followed by naturalistic treatment for 6.8 ± 0.8 months. All participants completed follow-up questionnaires; 28 completed follow-up fMRI. At each timepoint, three graph-theory network metrics of resting-state functional connectivity were computed: whole-brain weighted-degree (centrality), cortical integration (*between-network* connectivity), and cortical segregation (*within-network* connectivity). All analyses adjusted for age, sex, antidepressants, head motion, time between sessions and baseline score of interest, with cluster-wise correction. Results were contextualized against 50 age-, sex-, and head motion-matched healthy controls (HCs). Based on patient-reported Clinical Global Impression of Improvement ratings, 59.4% improved, 31.3% were unchanged, and 9.3% worsened. Core FND symptom (i.e. Screening for Somatoform Symptoms-7 Subscale for Conversion Disorder) and non-core physical symptom (Patient Health Questionnaire-15) scores showed variable trajectories, with no group-level changes. For whole-brain weighted-degree analyses, baseline centrality in right middle frontal, precentral, and left cerebellar regions was positively associated with core FND symptom change; longitudinally, centrality decreases in right precentral, superior parietal, lateral occipital, and cerebellar regions were associated with symptom improvement. For cortical integration analyses, baseline between-network connectivity in ventral attention, frontoparietal, and default mode network regions was positively associated with core FND symptom change; longitudinally, decreases in between-network connectivity for regions of these same networks were associated with symptom improvement. For cortical segregation analyses, baseline within-network connectivity in frontoparietal network regions was positively associated with core FND symptom change; no regions showed longitudinal segregation changes associated with symptom change. The right anterior insula emerged as a convergent site across baseline and longitudinal integration analyses, with the most improved participants showing elevated baseline between-network connectivity relative to HCs that normalized at follow-up. More modest functional connectivity associations were observed with non-core physical symptom change, spanning baseline within-network connectivity in dorsal attention network regions and longitudinal between-network connectivity increases in visual network regions. Findings remained significant adjusting for FND phenotype, although several attenuated when accounting for baseline affective symptoms or trauma burden. In conclusion, this study identified baseline and longitudinal resting-state functional connectivity features linked to symptom change in FND, highlighting the potential of large-scale network interactions as prognostic markers and providing mechanistic insights that set the stage for novel, biologically informed interventions.

## Teaser

Westlin *et al*. report that resting-state functional connectivity predicted and tracked with symptom change in functional neurological disorder over ∼6 months of naturalistic treatment. Greater improvement was linked to higher baseline integrated cortical connectivity and later reductions in ventral attention, frontoparietal, and default mode network regions—particularly the right anterior insula.

## Graphical Abstract

**Graphical Abstract:**
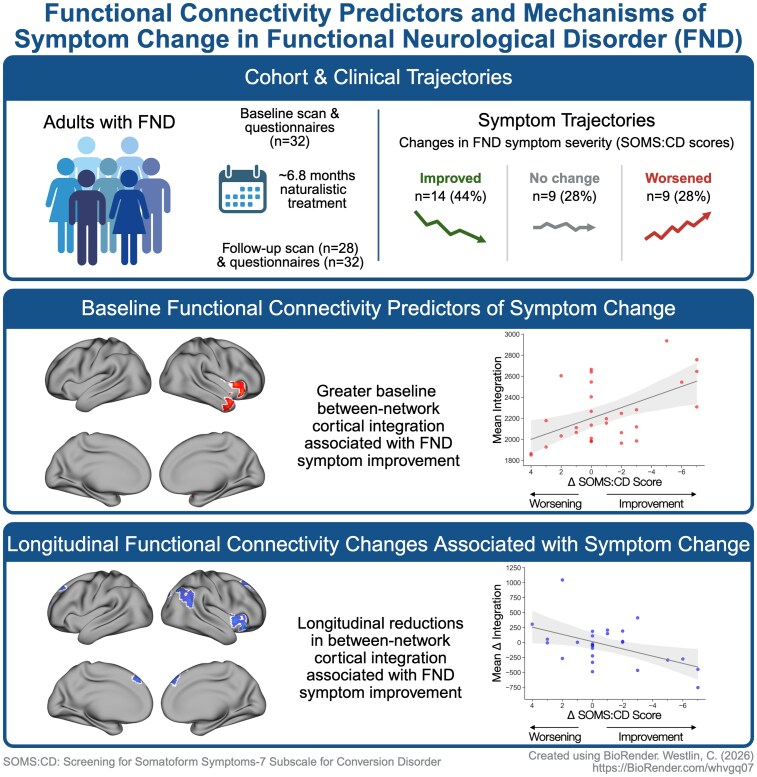

## Introduction

Functional neurological disorder (FND) is a common, costly, and potentially disabling condition at the neurology-psychiatry intersection, characterized by motor, sensory, and cognitive symptoms.^1-3^ Mixed symptoms occur in one-quarter to one-half of individuals, with others developing distinct functional neurological symptoms longitudinally.^4-6^ The most well-studied FND phenotypes are the motor (FND-motor) and seizure (FND-seizure) variants, frequently co-occurring with affective and trauma-related conditions.^5^ The biopsychosocial complexity of FND underscores the importance of multidisciplinary care, with clinical trials and naturalistic cohort studies supporting roles for psychotherapy and physical rehabilitation.^7-10^ Nonetheless, there is individual-level heterogeneity regarding clinical trajectories.^11-14^ As such, there is a need to investigate how brain network organization relates to FND symptom change over time.

Resting-state functional connectivity (rsFC) alterations in somatomotor (SMN), ventral attention (VAN)/salience, and default mode (DMN) networks, among others, have been identified across FND cohort studies.^15^ Several studies found aberrant connectivity between the VAN/salience network and motor control regions, and between the temporoparietal junction (TPJ) and sensorimotor regions.^16-21^ Work from our group identified altered functional network architecture in FND, marked by increased cortical integration (*between*-*network* connectivity) for SMN regions, with heightened connections to the VAN, DMN, and frontoparietal (FPN) networks.^22^ These findings support the hypothesis that distributed, network-level disruptions play a role in the pathophysiology of FND, with potential relevance as baseline predictors of clinical trajectories and as mechanisms underlying longitudinal symptom change.

Research has begun to explore neuroimaging correlates of clinical trajectories in FND.^17,21,23-26^ For example, improvements in functional tremor following cognitive behavioural therapy (CBT) were linked to decreased anterior cingulate and paracingulate activations.^24^ Following 1 week of multidisciplinary treatment, increased amygdala-ventromedial prefrontal cortex functional connectivity was observed in the subset of FND-motor patients with a favourable response.^25^ Improved outcomes in a mixed FND cohort have been associated with greater supplementary motor area signal variability, whereas worse outcomes were associated with higher baseline left insula variability.^26^ In earlier FND research, we identified an association between improved symptom scores and relative baseline increases in left centromedial amygdala-to-right anterior insula rsFC.^17^ Nonetheless, much of the literature has relied on region-of-interest (ROI) approaches, examined either baseline predictors or post-treatment mechanisms in isolation, or focused on narrowly defined subpopulations (e.g. euthymic individuals only^23^). This underscores the need for longitudinal, whole-brain investigations that examine both predictors and mechanisms of symptom change in a representative FND cohort.

Here, we employed rsFC graph-theory analyses to examine baseline predictors (*n* = 32) and longitudinal mechanisms (*n* = 28) of symptom change in FND. Patients underwent baseline and 6-month follow-up neuroimaging, between which they received individualized, naturalistic treatment as part of usual care. At both timepoints, we assessed three network organization metrics: whole-brain weighted-degree (WD; centrality), cortical integration (*between-network* connectivity), and cortical segregation (*within-network* connectivity). Our goal was to identify baseline brain network features, and longitudinal changes in network organization, that related to individual differences in symptom change. By linking brain architecture to clinical trajectories, this work aims to advance FND mechanistic models and guide the future development of personalized, neurobiologically informed interventions.

## Materials and methods

### Participants

Thirty-two consecutive participants with FND [28 females, 4 males; mean age = 42.4 ± 13.3; average illness duration 3.7 ± 4.2 years (range = 0.1–16.1 years)] were recruited from the Massachusetts General Hospital FND Unit between March 2021 and October 2024 (Table 1). FND diagnoses were based on positive examination signs, semiological features, and electroencephalography data (FND-seizure only^27^). Participants with either FND-motor, FND-seizure, or both were eligible, reflecting observations that individuals often present with mixed symptoms and/or develop new FND symptoms over time.^5^ Exclusion criteria included major neurological comorbidities (e.g. epilepsy, Parkinson’s disease), known brain MRI abnormalities, poorly controlled medical conditions with central nervous system (CNS) effects, active illicit substance dependence, psychosis, and/or active suicidality. Twenty-seven had FND-motor (tremor = 12; gait = 12; speech = 12; weakness = 8; tics/jerks/spasms = 5; dystonia = 2; presentations were not mutually exclusive) and 10 had FND-seizure (documented = 6; probable = 4); 5 participants had FND-motor and FND-seizure (see Supplementary Fig. 1 for a depiction of FND symptoms across participants). Only cross-sectional (baseline) neuroimaging data for this cohort have been previously published.^22,28,29^ Twenty-eight of 32 individuals fully completed the follow-up session (four provided follow-up questionnaire data only; see Supplementary Table 1 sub-cohort demographics). One participant changed selective serotonin reuptake inhibitor (SSRI)/serotonin noradrenaline reuptake inhibitor (SNRI) use status during the study period and was included only in predictor analyses; SSRI/SNRI use remained stable for all participants included in longitudinal analyses.

**Table 1:** Clinical characteristics of the longitudinal functional neurological disorder cohort

	Baseline (*n* = 32) mean ± SD or *N* (%)	Follow-up (*n* = 32) mean ± SD or *N* (%)	Uncorrected *P*-value^a^
Characteristics			
Age at consent (yrs.)	42.4 ± 13.3; range: 22.4–72.1; median: 44.0		
Biological sex	28F; 4M		
Illness duration (yrs.)	3.7 ± 4.2; range: 0.1–16.1; median: 2.0		
Interval follow-up period (months)	6.8 ± 0.8; range 5.4–8.8; median 6.6		
Race (white)	28 (87.5%)		
Married	15 (46.9%)	15 (46.9%)	1.00
College graduate	22 (68.8%)		
Employed full-time (or full-time student)	12 (37.5%)	14 (43.8%)	0.69
On medical disability or worker’s compensation	10 (31.3%)	6 (18.8%)	0.25
Functional motor disorder	27 (84.4%)		
Functional seizures	10 (31.3%)		
SSRI/SNRI use	13 (40.6%)	14 (43.8%)	1.00
Framewise displacement	0.07 ± 0.04; range: 0.02–0.2; median: 0.06	0.08 ± 0.03; range: 0.02–0.1; median: 0.08^b^	0.68^c^
Psychometric Scores			
CGI-I		Improved^d^: 19 (59.4%) No change: 10 (31.3%) Worsened: 3 (9.3%)	
SOMS:CD	6.6 ± 6.3; range: 0–21; median: 5	5.7 ± 5.8; range: 0–22; median: 4.5	0.21
PHQ-15	11.8 ± 5.7; range: 1–26; median: 11.0	12.2 ± 5.7; range: 3–25; median: 12.0	0.55
BDI-II	15.3 ± 11.4; range: 0–38; median: 13.0	12.1 ± 11.3; range 0–40; median: 9.5	0.018
STAI-total	82.5 ± 23.2; range 49–140; median: 77.5	77.3 ± 21.2; range 44–120; median 78.5	0.038
PCL-5	23.4 ± 17.9; range: 0–62; median: 20		
CTQ-abuse	31.0 ± 14.7; range: 15–68; median: 27.5		
CTQ-neglect	21.6 ± 8.5; range: 10–37; median: 19.5		

SSRI/SNRI, selective serotonin reuptake inhibitor/serotonin noradrenaline reuptake inhibitor; SOMS:CD, Screening for Somatoform Symptoms-7 Subscale for Conversion Disorder; PHQ-15, Patient Health Questionnaire-15; BDI-II, Beck Depression Inventory-II; STAI, Spielberger State-Trait Anxiety Inventory; PCL-5, PTSD Checklist 5; CTQ, Childhood Trauma Questionnaire.

^a^ *P*-value corresponds to uncorrected statistic for baseline versus follow-up comparisons. For psychometric scores, FDR-corrected *P*-values are as follows: SOMS:CD: 0.28, PHQ-15: 0.55, BDI-II: 0.072, STAI-total: 0.076. For characteristic variables, McNemar tests were used to assess married, employed, medical disability, and SSRI/SNRI use variables; for psychometric score variables, paired samples *t*-tests were used to assess pre-post changes for PHQ-15 and STAI-total scores (normally distributed variables), and Wilcoxon signed-rank tests were used for SOMS:CD and BDI-II (non-normally distributed).

^b^Framewise displacement for the follow-up cohort is reported for the 28 participants who completed MRI scanning.

^c^A paired sample *t*-test was conducted between the 28 participants who completed both baseline and follow-up MRI scanning.

^d^Improved includes: ‘much improved’ (*n* = 5) and ‘improved’ (*n* = 14) based on patient-reported Clinical Global Impression of Improvement (CGI-I) scale scores.

Five additional participants were excluded following imaging acquisition and processing due to excessive head motion (*n* = 4) or following outlier inspection based on mean whole-brain WD (*n* = 1) as previously described.^22^ Twenty additional participants provided baseline psychometric and imaging data but did not complete at least the follow-up questionnaires (Supplementary Table 2). These participants were classified as lost to research follow-up if they did not complete the follow-up session after at least two contact attempts.

Fifty healthy controls [HCs, 43 females, 7 males; mean age = 38.7 ± 10.5 years; mean head motion (framewise displacement) = 0.06 ± 0.03] were also included for *post hoc* comparisons. These participants were prospectively recruited from the community and had no psychiatric, major neurological, or poorly controlled medical conditions with CNS effects. HCs were matched to the FND cohort based on age, sex, and head motion. All participants provided informed consent, and the Mass General Brigham Institutional Review Board approved this study.

### Study design and individualized, naturalistic treatment

At baseline, participants had an MRI scan, underwent a Structured Clinical Interview (SCID-DSM-5), and completed questionnaires. Approximately 6 months later (mean 6.8 ± 0.8 months), participants returned for a repeat MRI scan and follow-up questionnaires.

Between baseline and follow-up sessions, participants received naturalistic, individualized treatment (usual care at our institution) as determined collaboratively by the patient and FND Unit team members. All participants remained in longitudinal care with an FND Unit neurologist or neuropsychiatrist. As increasingly endorsed,^30^ treatment recommendations were individualized based on: (i) phenotype, (ii) concurrent neuropsychiatric and psychosocial factors,^7^ (iii) published recommendations,^31-33^ (iv) original research protocols^8,34^—including treatment studies from our own group,^35,36^ (v) patient preferences, and (vi) travel and insurance considerations among other factors. Interventions included psychotherapy, physiotherapy, occupational therapy, and/or speech and language therapy. Treatment was not limited to our healthcare system, and thus a subset received care across our academic medical centre and the community.

### Neuropsychiatric characterization and longitudinal monitoring

At baseline, participants completed questionnaires to assess symptom severity and other neuropsychiatric features. Core FND symptom burden was evaluated using the Screening for Somatoform Symptoms-7 Subscale for Conversion Disorder (SOMS:CD), a questionnaire of FND symptoms (e.g. paralysis, impaired balance, seizures) experienced during the past week, rated on a 5-point Likert scale.^37^ Non-core physical symptom severity was assessed with the Patient Health Questionnaire-15 (PHQ-15), which captures bothersome physical symptoms (e.g. pain, fatigue) during the previous four weeks, using a 3-point Likert scale.^38^ Participants also completed the Beck Depression Inventory-II (BDI-II), Spielberger State-Trait Anxiety Inventory (STAI), PTSD Checklist-5 (PCL-5), and the Childhood Trauma Questionnaire (CTQ). At follow-up, participants completed the SOMS:CD, PHQ-15, BDI-II, and STAI, along with laboratory-designed questions to assess the treatments received in the interval period (see Supplementary Materials); participants also completed the Clinical Global Impression of Improvement scale (CGI-I) to evaluate their perceived change in FND clinical status, based on a 5-point Likert scale (‘much improved’ to ‘much worse’).

### MRI acquisition and preprocessing

All participants were scanned on the same Siemens Tim Trio 3T MRI scanner using a 12-channel phased-array head coil. A high-resolution T1-weighted magnetization-prepared rapid gradient echo scan was acquired for each subject with the following parameters: 1 mm isotropic voxels; 160 sagittal slices; acquisition matrix size = 256 × 256; repetition time = 2300 ms; echo time = 2.98 ms; field of view = 256 mm. Resting-state blood-oxygen-level-dependent (BOLD) functional scans were acquired using T2*-weighted echo-planar imaging sequences with the following parameters: TR = 3000 ms; TE = 30 ms; flip angle 85°; 216 mm FOV; 3 mm isotropic voxels; sequence length = 6 min, 12 s. All participants had two resting-state BOLD acquisitions. Participants were instructed to remain as still as possible with their eyes open, bi-temporal foam pads restricted head motion, and earplugs were used to attenuate scanner noise.

Preprocessing for anatomical and functional MRI data was done using FMRIB Software Library v5.0.7 (FSL, Oxford, UK) and MATLAB 2023a (MathWorks, Natick, MA, USA) with in-house preprocessing pipelines as described previously.^22,39^ Preprocessing of T1-weighted anatomical images included: reorientation to right-posterior-inferior (RPI); alignment to the anterior and posterior commissures; skull stripping; segmentation of grey matter, white matter, and cerebrospinal fluid; and computation of a non-linear transformation between individual skull-stripped T1 images and a 3 mm resolution MNI152 template. fMRI preprocessing steps included: discarding the first four volumes; slice timing correction; reorientation to RPI; realignment of functional volumes via a 6-parameter rigid body transformation; computation of the transformation between individual skull-stripped T1 images and mean functional images; intensity normalization; and regression of nuisance signals, including 12 motion-related covariates (rigid motion parameters and their derivatives), linear and quadratic terms, and five components each from the lateral ventricles and white matter. Further steps included transformation to MNI space, spatial smoothing using a 6 mm full-width at half-maximum Gaussian kernel, and temporal band-pass filtering (0.01–0.08 Hz). Head motion was quantified using realignment parameters, including three translation and three rotation estimates, and volumes with excessive head motion (framewise displacement >0.5 mm) were removed from the data. Analyses were performed on 120 time points per subject; resting-state acquisition runs were concatenated, and the first 120 time points were retained to standardize the time series length across participants.

### Resting-state functional connectivity

**Figure 1:**
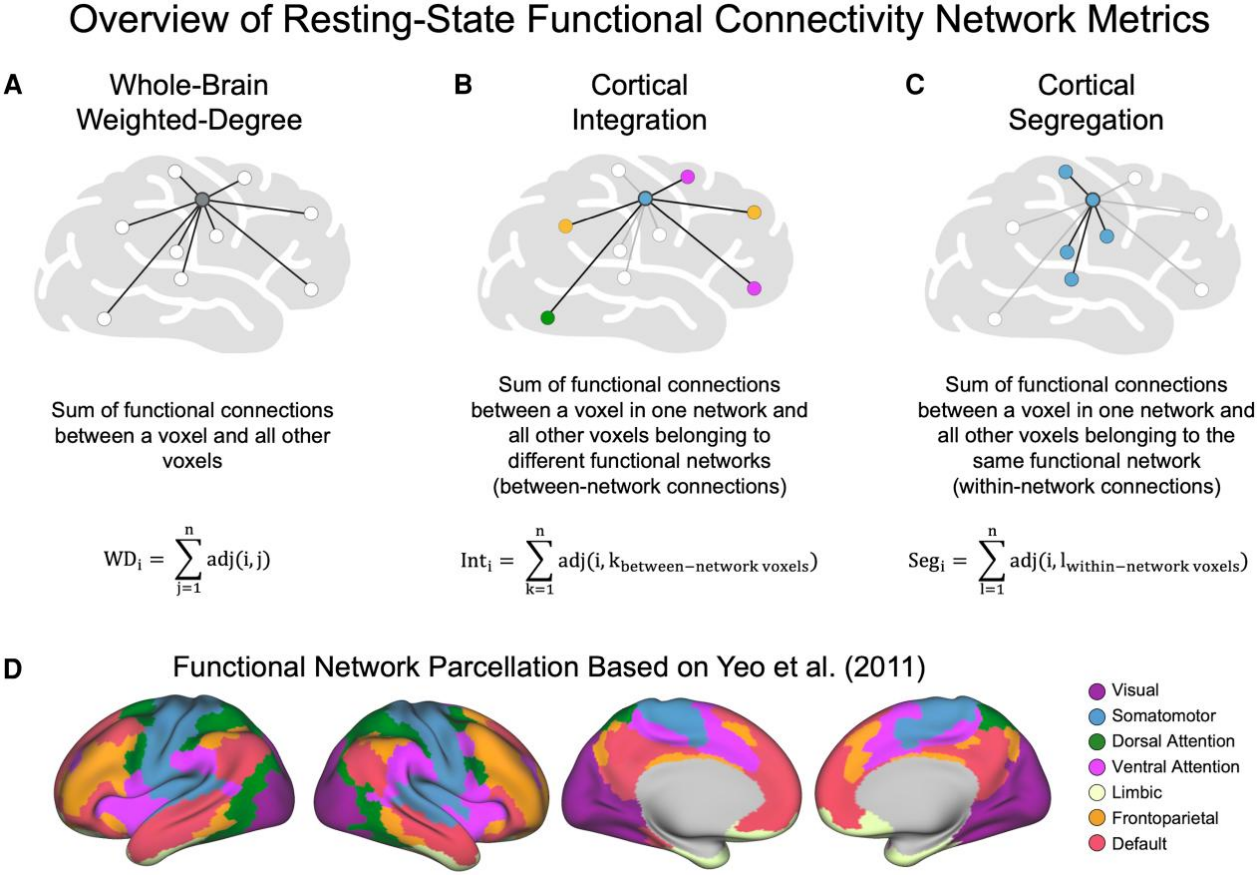
**Overview of graph theory-based resting-state functional connectivity metrics used in the present study.** (**A**) Whole-brain weighted-degree is computed by summing the weighted connections for a given voxel and all other voxels. A higher weighted-degree therefore indicates that a given voxel is more globally connected to the rest of the brain. (**B**) Cortical integration is computed by summing the cortical weighted connections for a given voxel in one network and all other voxels belonging to a *different* functional network than the originating voxel. A high integration value therefore indicates that a given voxel is important for between-network communication. (**C**) Cortical segregation is computed by summing the cortical weighted connections for a given voxel in one network and all other voxels belonging to the *same* network. A high segregation value therefore indicates that a given voxel is important for within-network modular communication. For each of these metrics, only positive connections were summed. (**D**) Visualization of the Yeo *et al*. seven-network parcellation used to assign voxels to resting-state networks in integration and segregation analyses. Portions of this figure were adapted from a prior open access article.^22^

Voxel-wise network properties were quantified using three graph theory metrics—whole-brain WD, cortical integration, and cortical segregation—as previously described^22^; see Fig. 1 for an overview. rsFC matrices were computed per participant by calculating Pearson correlation coefficients between the time series of each pair of grey matter voxels. Correlation coefficients were Fisher r-to-z transformed prior to calculation of graph theory metrics and negative correlations were removed due to their controversial interpretation.

WD was calculated from whole-brain rsFC matrices to assess the global connectivity of each voxel. For a given voxel *i*, WD was defined as the sum of its weighted positive connections:


(1) $$WD_{i}=\;\overset{n}{\underset{\;j=1}{\sum }}\;\mathrm{adj}(i,\;j)$$

where adj(*i, j*) is the weighted functional connection between voxels *i* and *j*. The result was a subject-level map indicating how strongly each voxel was connected to the rest of the brain.

Integration and segregation metrics were computed from cortical-only rsFC matrices, in which each voxel was assigned to one of seven functional networks based on Yeo *et al*.: SMN, dorsal attention (DAN), VAN, FPN, DMN, limbic (LIM), and visual (VIS) networks.^40^ Connections between voxels in *different* networks were categorized as integration connections, whereas connections within the *same* network were labelled as segregation connections. For each voxel *i*, integration and segregation were defined as:


(2) $$\mathrm{Integratio}n_{i}=\;\overset{n}{\underset{k=1}{\sum }}\;\mathrm{adj}(i,k_{\mathrm{between}\text{-}\mathrm{network}\;\mathrm{voxels}})$$

(3) $$\mathrm{Segregatio}n_{i}=\;\overset{n}{\underset{l=1}{\sum }}\;\mathrm{adj}(i,l_{\mathrm{within}\text{-}\mathrm{network}\;\mathrm{voxels}})$$

These calculations each yielded a voxel-wise map per participant, with the cortical integration map reflecting the extent to which each voxel integrated across networks, and the cortical segregation map reflecting its within-network connectivity.

### Statistical analyses

#### Baseline rsFC predictors of symptom change

Symptom change was computed as the difference between follow-up and baseline scores for core FND symptoms (SOMS:CD), and separately for non-core physical symptoms (PHQ-15). Separate models were run for each rsFC measure (whole-brain WD, cortical integration, and cortical segregation) and symptom change scores. All general linear models (GLMs) controlled for age, sex, SSRI/SNRI use (yes/no), mean FD, time between baseline and follow-up, and the baseline symptom score of interest (SOMS:CD or PHQ-15). Findings were corrected for multiple comparisons using cluster-wise correction via Monte Carlo simulation with 10 000 iterations, estimating the probability of observing false positive clusters at *P* < 0.05. *Post hoc* analyses were conducted to assess whether findings remained significant adjusting for: (i) FND phenotype (FND-seizure yes/no); (ii) baseline BDI-II, STAI-total, and PCL-5 scores; and (iii) CTQ-abuse and CTQ-neglect scores.

#### Longitudinal changes in rsFC associated with symptom change

To examine whether longitudinal changes in rsFC network organization were associated with clinical trajectories, we conducted the same series of voxel-wise GLMs described in the above baseline predictors section, using updated independent variables. Specifically, the independent variables were rsFC change maps for each network measure (whole-brain WD, cortical integration, cortical segregation), computed as the difference between follow-up and baseline rsFC maps. These GLM analyses included the same primary and *post hoc* adjustments as described above, with two additional *post hoc* adjustments: (i) change in BDI-II scores and (ii) change in STAI-total scores. No participants in this analysis had a change in SSRI/SNRI use (yes/no). Participants did not differ based on mean FD between baseline and follow-up (*P* = 0.68).

### *Post hoc* analyses to characterize cortical integration findings

To understand which functional networks the voxels with significant cortical integration-symptom change associations were integrating across, we conducted *post hoc* seed-based connectivity analyses. Specifically, we assigned each voxel that showed a significant association between integration-related rsFC and symptom change to one of the seven Yeo *et al*. networks, and created a seed ROI for each network by averaging the time series across all voxels within that network. For the top three networks containing the most significant voxels, we then computed Pearson correlations between the average time series of the seed and the time series of all cortical voxels, and transformed these values using Fisher’s r-to-z transformation. The resulting subject-level connectivity maps were entered into voxel-wise GLMs, testing whether rsFC values significantly differed from zero while adjusting for age, sex, SSRI/SNRI use, and mean FD. Results were corrected for multiple comparisons using cluster-wise correction via Monte Carlo simulation with 10 000 iterations to estimate the probability of false positive clusters with *P* < 0.05.

To quantify the network-level connectivity patterns, each significant cortical voxel in the resulting connectivity maps was labelled according to the Yeo *et al*. seven-network parcellation. We then computed the number of significant connections from each seed network to each of the six other functional networks (excluding within-network links that are not relevant for measuring integration).

### *Post hoc* contextualization with HCs

To further contextualize findings, we conducted an exploratory comparison with HCs. We identified overlapping voxels that emerged in *both* baseline predictor and longitudinal change analyses, and extracted mean values across these voxels in HCs and in FND participants at baseline and follow-up. Mann–Whitney U-tests were used to compare mean values of HCs versus FND participants, across the full FND cohort and stratified by the top/bottom 20% of individuals who reported the greatest symptom improvement/worsening. To further test the specificity of our longitudinal findings, exploratory *post hoc* analyses were also conducted stratifying participants by BDI-II or STAI-total change scores, to examine whether similar patterns were observed when participants were grouped according to changes in depression or anxiety symptoms.

## Results

### Clinical observations

Among the 32 participants, 30 (94%) received psychotherapy, with 21 (65.6%) specifically reporting receipt of CBT. Seventeen participants (53%) reported receiving physiotherapy, 16 (50%) endorsed receiving occupational therapy, and 10 (31%) reported receiving speech and language therapy. Eleven participants (34.4%) had all FND care within our healthcare system; the remainder either received a combination of treatment within our healthcare system and the community (*n* = 16, 50.0%) or entirely in the community (*n* = 5, 15.6%), limiting our ability to objectively capture treatment-related frequency and duration information (Table 1 and Supplementary Table 3).

Based on CGI-I scores, 5 participants (15.6%) reported being ‘much improved’, 14 (43.8%) ‘improved’, 10 (31.3%) ‘unchanged’, and 3 (9.3%) ‘worse’; no participants rated themselves as ‘much worse’. SOMS:CD and PHQ-15 scores showed no group-level differences at follow-up versus baseline (Wilcoxon signed-rank tests: SOMS:CD: *z* = −1.25, *P* = 0.21; PHQ-15: *z* = −0.43, *P* = 0.67). However, scores showed prominent individual differences. For SOMS:CD scores, 14 participants reported reduced symptoms (mean change = −4.4 ± 3.3; median = −3), 9 reported no change, and 9 endorsed increased symptoms (mean change = 3.9 ± 4.3; median = 3). For PHQ-15 scores, 15 participants reported reduced symptoms (mean change = −2.6 ± 1.2; median = −3), 1 reported no change, and 16 endorsed increased symptoms (mean change = 3.1 ± 1.8; median = 3) (Table 1).

No baseline demographic or psychometric variables were associated with symptom improvement (SOMS:CD change scores; PHQ-15 change scores; CGI-I improved versus not-improved) following false discovery rate correction at the level of each symptom measure. However, at an uncorrected threshold (*P* < 0.05), those who reported improvement on the CGI-I had shorter illness durations (3.2 ± 4.7 years versus 4.5 ± 3.4 years) and were less likely to have FND-seizure (Supplementary Table 4). No baseline characteristics showed uncorrected associations with SOMS:CD or PHQ-15 change scores.

Compared to longitudinal cohort participants (*n* = 32), those lost to follow-up (*n* = 20) showed no significant between-group differences on available baseline characteristics following false discovery rate correction (Supplementary Table 2).

### Baseline functional connectivity predictors of symptom change

#### Whole-brain WD

**Figure 2:**
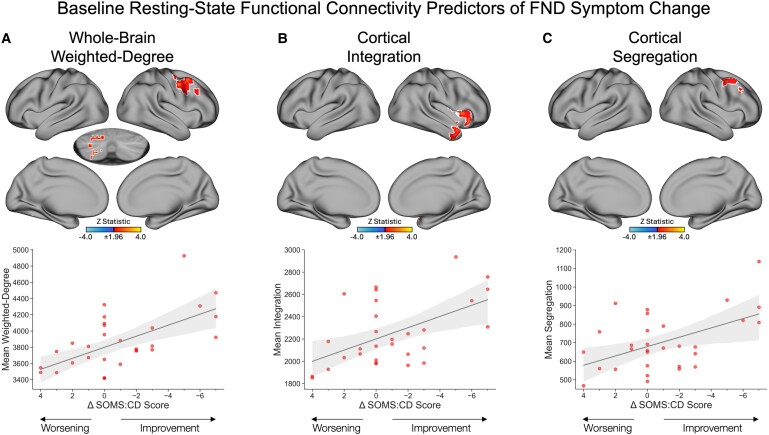
**Baseline resting-state functional connectivity predictors of functional neurological disorder (FND) symptom change, as measured by a change in SOMS:CD scores between baseline and follow-up (*N* = 32).** (**A**) *Top* panel depicts regions with baseline whole-brain weighted-degree values that were significantly associated with FND symptom change. Specifically, baseline WD in the right middle frontal gyrus, right precentral gyrus, and left cerebellum was positively associated with FND symptom change, such that a higher baseline weighted-degree in these regions was associated with greater symptom improvement, and a lower weighted-degree was associated with a greater worsening of symptoms. *Bottom* panel depicts a scatterplot of SOMS:CD change scores on the *x*-axis and mean weighted-degree values averaged across significant voxels on the *y*-axis. (**B**) *Top* panel depicts a positive association between baseline cortical integration values in the right insula and right temporal pole and FND symptom change scores. Increased baseline integration in these regions was associated with symptom improvement, while decreased baseline integration was associated with symptom worsening. *Bottom* panel depicts a scatterplot of SOMS:CD change scores on the *x*-axis and mean integration values averaged across significant voxels on the *y*-axis. (**C**) *Top* panel depicts a positive association between baseline cortical segregation values in the right middle frontal and precentral gyri and SOMS:CD change scores, such that increased segregation in these regions was associated with symptom improvement, and decreased segregation was associated with symptom worsening. *Bottom* panel depicts a scatterplot of SOMS:CD change scores on the *x*-axis and mean segregation values averaged across significant voxels on the *y*-axis. Colours in the maps reflect the *z*-statistic for the primary general linear model adjusting for age, sex, SSRI/SNRI use, mean framewise displacement, elapsed time between baseline and follow-up sessions, and baseline SOMS:CD score (*z*-statistic > 1.96; *P* < 0.05 cluster-corrected for multiple comparisons). White outlines reflect regions that also held across *post hoc* corrections for FND phenotype (FND-seizure yes/no). Statistical maps for additional *post hoc* corrections are visualized in Supplementary Fig. 3. Subcortical images are shown in neurological view (left = left). Analyses were conducted at the voxel-wise level, scatterplots of mean values across voxels are included for illustrative purposes. Datapoints in each scatterplot reflect the mean weighted-degree/integration/segregation value and corresponding SOMS:CD change score for each individual participant. Shaded regions reflect 95% confidence intervals. For visualization purposes, change scores are plotted with negative values on the right to reflect symptom improvement. Additionally, two participants with SOMS:CD change scores that exceeded more than 1.5 times the interquartile range above the upper quartile or below the lower quartile were excluded from the plots for clarity, but retained in analyses. SOMS:CD, Screening for Somatoform Symptoms-7 Subscale for Conversion Disorder; SSRI/SNRI, selective serotonin reuptake inhibitor/serotonin noradrenaline reuptake inhibitor.

Baseline rsFC WD values in the right middle frontal and precentral gyri, and left cerebellum, were positively associated with changes in SOMS:CD scores at follow-up compared to baseline (Fig. 2A). Within the cerebellum, findings were localized to the left hemisphere and spanned lobules VI, CrusI/II, VIIb, VIIa/VIIb, and X (Supplementary Fig. 2). Specifically, higher baseline WD in these areas was linked to greater FND symptom improvement, while lower baseline WD in these regions related to symptom worsening. All findings held adjusting *post hoc* for phenotype (FND-seizure yes/no), but only the cerebellar findings remained significant for CTQ-abuse and CTQ-neglect scores; no findings held adjusting for baseline BDI-II, STAI-total, or PCL-5 scores (Supplementary Fig. 3). No regions showed an association between baseline WD values and PHQ-15 score changes.

#### Cortical integration

Baseline integration values in the right anterior insula and temporal pole were positively associated with changes in SOMS:CD scores. Specifically, higher baseline integration in these regions related to longitudinal FND symptom improvement, and lower baseline integration values related to symptom worsening (Fig. 2B). These findings held when adjusting *post hoc* for phenotype (FND-seizure yes/no) but did not hold when adjusting for baseline affective symptoms or childhood trauma (Supplementary Fig. 3). For an additional inquiry into the discrete impact of the five trauma domains of the CTQ, see Supplementary Fig. 4. No regions showed an association between baseline integration values and PHQ-15 score changes.

#### *Post hoc* characterization of baseline integration-based network connectivity

**Figure 3:**
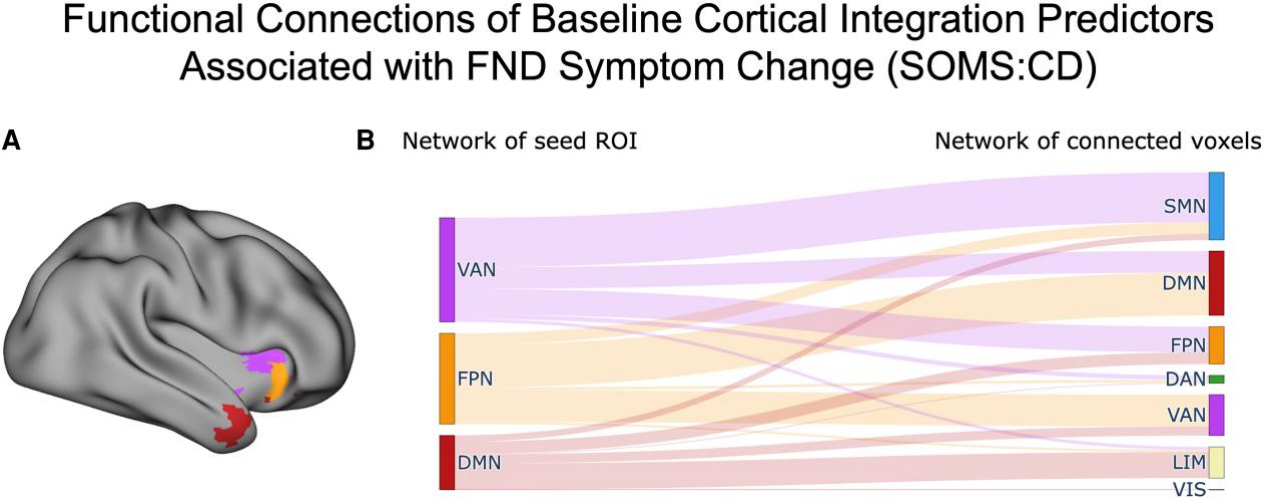
**Network connectivity of baseline cortical integration predictors associated with functional neurological disorder (FND) symptom change (*N* = 32).** (**A**) Depicts voxels whose baseline integration values were significantly associated with FND symptom change (ΔSOMS:CD scores), coloured according to their functional network assignment based on the Yeo *et al*. seven-network parcellation. Only the three networks containing the most significant voxels are displayed: ventral attention (VAN; magenta), frontoparietal (FPN; orange), and default mode network (DMN; red). For each of these networks, we extracted the average time series of all significant voxels for use in seed-to-voxel connectivity analyses, to then characterize the networks they were functionally integrating across. (**B**) Depicts a Sankey diagram summarizing the between-network connectivity patterns for each seed. The left side of the diagram denotes the seed network, while the right side indicates the network identity of significantly connected voxels. Line thickness reflects the number of significant connections, with thicker lines indicating a higher number of between-network connections. Self-connections (e.g. DMN-to-DMN) were excluded, as integration analyses focused on between-network connectivity. See Supplementary Fig. 5 for detailed connectivity breakdowns across all networks. SMN, somatomotor network; DMN, default mode network; FPN, frontoparietal network; DAN, dorsal attention network; VAN, ventral attention network; LIM, limbic network; VIS, visual network; SOMS:CD, Screening for Somatoform Symptoms-7 Subscale for Conversion Disorder.

To investigate what networks were connected to the regions that showed associations between baseline cortical integration and SOMS:CD change scores, we conducted *post hoc* seed-to-voxel connectivity analyses. Most of the identified voxels were within VAN, FPN, and DMN. The VAN seed at baseline showed increased rsFC largely to SMN, FPN, and DMN regions, while the FPN seed showed increased connectivity largely to DMN, VAN, and SMN regions; the DMN seed showed increased connectivity largely to LIM, FPN, VAN, and SMN (Fig. 3; Supplementary Fig. 5).

#### Cortical segregation

Baseline segregation values in FPN regions of the right middle frontal and precentral gyri were positively associated with a longitudinal change in SOMS:CD scores, such that symptom improvement was linked to higher baseline segregation connections, and symptom worsening was linked to lower baseline segregation connections (Fig. 2C). These findings held when adjusting *post hoc* for phenotype (FND-seizure yes/no), and partially held when adjusting for baseline BDI-II, STAI-total, and PCL-5 scores; findings did not hold when adjusting for CTQ-abuse and CTQ-neglect scores (Supplementary Fig. 3).

**Figure 4:**
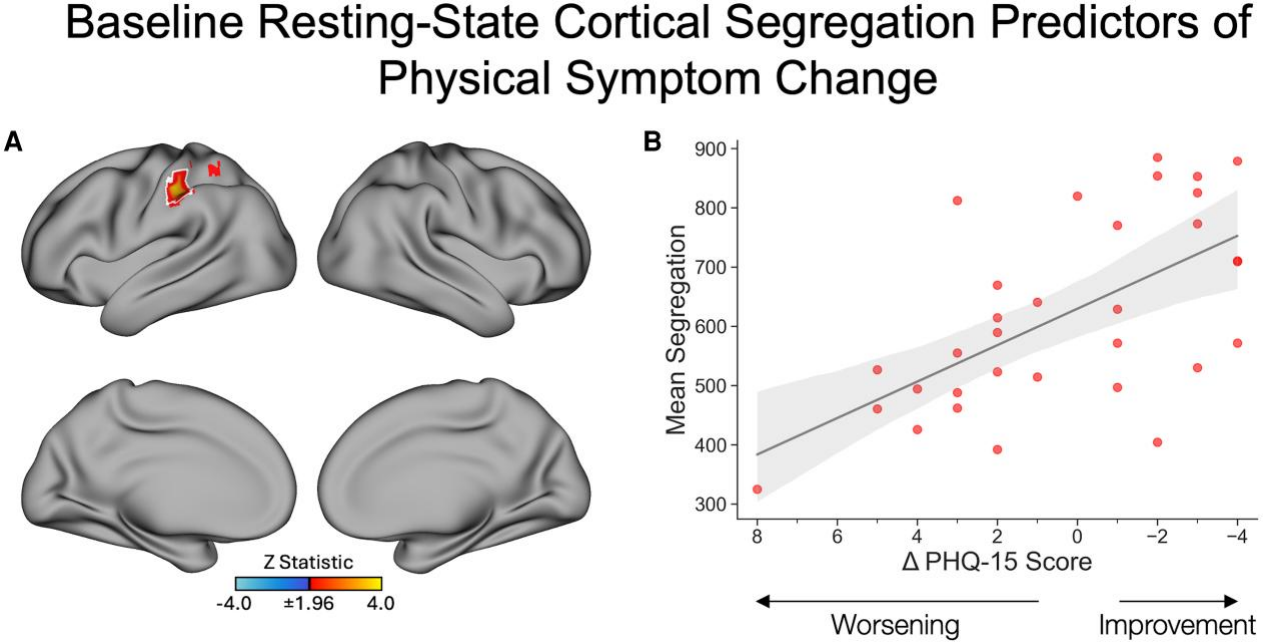
**Baseline resting-state cortical segregation predictors of physical symptom change, as measured by a change in PHQ-15 scores between baseline and follow-up (*N* = 32).** (**A**) Depicts regions with baseline segregation values that were significantly associated with changes in self-reported physical symptoms. Specifically, baseline segregation in the left post-central and left supramarginal gyri was positively associated with physical symptom change, such that a higher baseline segregation in these regions was associated with greater symptom improvement, and a lower segregation was associated with a greater worsening of symptoms. Colours in the map reflect the *z*-statistic for the primary GLM adjusting for age, sex, SSRI/SNRI use, mean framewise displacement, elapsed time between baseline and follow-up sessions, and baseline PHQ-15 score (*z*-statistic > 1.96; *P* < 0.05 cluster-corrected for multiple comparisons). White outlines reflect regions that also held across *post hoc* correction for FND phenotype (FND-seizure yes/no). Statistical maps for additional *post hoc* corrections are visualized in Supplementary Fig. 6. (**B**) Depicts a scatterplot of PHQ-15 change scores on the *x*-axis and mean segregation values averaged across significant voxels on the *y*-axis. Datapoints in the scatterplot reflect the mean segregation value and corresponding PHQ-15 change score for each individual participant. The shaded region reflects the 95% confidence interval. For visualization purposes, change scores are plotted with negative values on the right to reflect symptom improvement. PHQ-15, Patient Health Questionnaire-15.

Baseline segregation values in the left post-central and supramarginal gyri of the DAN were associated with a change in PHQ-15 scores, with an increase in baseline segregation linked to physical symptom improvement at follow-up, and a decrease in baseline segregation linked to physical symptom worsening (Fig. 4). Findings held across *post hoc* adjustments for (i) phenotype (FND-seizure yes/no) and (ii) baseline BDI-II, STAI-total, and PCL-5 scores; findings did not remain significant adjusting *post hoc* for CTQ-abuse and CTQ-neglect scores (Supplementary Fig. 6).

### Longitudinal changes in functional connectivity associated with symptom change

#### Whole-brain WD

**Figure 5:**
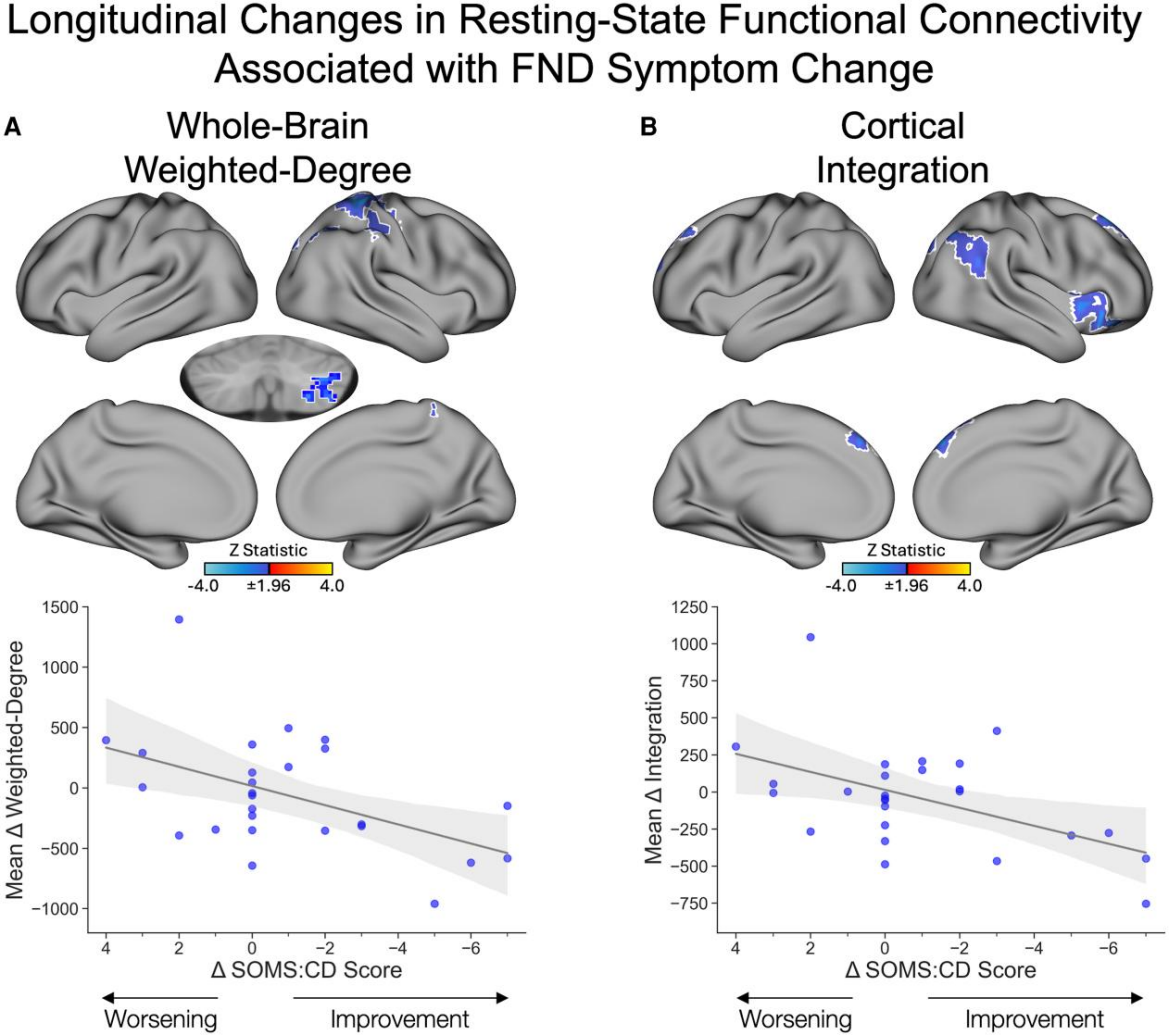
**Longitudinal changes in resting-state functional connectivity associated with functional neurological disorder (FND) symptom change (ΔSOMS:CD scores) between baseline and follow-up (*N* = 28).** (**A**) *Top* panel depicts regions with changes in whole-brain weighted-degree that were significantly negatively associated with FND symptom change. Specifically, symptom improvement was associated with decreased weighted-degree at follow-up compared to baseline in the right lateral occipital cortex, superior parietal lobule, precentral gyrus, and cerebellum, while increased weighted-degree in these regions at follow-up compared to baseline was associated with symptom worsening. *Bottom* panel depicts a scatterplot of SOMS:CD change scores on the *x*-axis and mean weighted-degree change values averaged across significant voxels on the *y*-axis. (**B**) *Top* panel depicts a negative association between cortical integration change and FND symptom change between baseline and follow-up. Symptom improvement was associated with decreased integration at follow-up compared to baseline in the right anterior insula and orbitofrontal cortex, angular gyrus, lateral occipital cortex, and bilateral frontal pole and superior frontal gyrus, and symptom worsening was associated with increased integration at follow-up compared to baseline in these regions. *Bottom* panel depicts a scatterplot of SOMS:CD change scores on the *x*-axis and mean integration change values averaged across significant voxels on the *y*-axis. Colours in the maps reflect the *z*-statistic for the primary general linear model adjusting for age, sex, SSRI/SNRI use, mean framewise displacement, elapsed time between baseline and follow-up sessions, and baseline SOMS:CD score (*z*-statistic > 1.96; *P* < 0.05 cluster-corrected for multiple comparisons). White outlines reflect regions that also held across *post hoc* corrections for FND phenotype (FND-seizure yes/no). Statistical maps for additional *post hoc* corrections are visualized in Supplementary Fig. 7. Subcortical images are shown in neurological view (left = left). Analyses were conducted at the voxel-wise level, scatterplots of mean values across voxels are included for illustrative purposes. Datapoints in each scatterplot reflect the mean weighted-degree/integration/segregation change value and corresponding SOMS:CD change score for each individual participant. Shaded regions reflect 95% confidence intervals. For visualization purposes, change scores are plotted with negative values on the right to reflect symptom improvement. Additionally, two participants with SOMS:CD change scores that exceeded more than 1.5 times the interquartile range above the upper quartile or below the lower quartile were excluded from the plots for clarity, but retained in analyses. SOMS:CD, Screening for Somatoform Symptoms-7 Subscale for Conversion Disorder; SSRI/SNRI, selective serotonin reuptake inhibitor/serotonin noradrenaline reuptake inhibitor.

Longitudinal rsFC WD changes in the right precentral gyrus, superior parietal lobule, lateral occipital cortex, and cerebellum were negatively associated with SOMS:CD symptom changes between follow-up and baseline (Fig. 5A). Within the cerebellum, findings were localized to the right hemisphere and spanned lobules VI, CrusI/II, VIIb, and VIIa/VIIb (Supplementary Fig. 2). Specifically, as WD values decreased at follow-up compared to baseline, symptom scores improved. Cerebellar findings held across all *post hoc* adjustments. Cortical findings held across *post hoc* adjustments for (i) phenotype (FND-seizure yes/no); (ii) CTQ-abuse and CTQ-neglect scores; (iii) BDI-II score change; and (iv) STAI-total score change but did not remain significant when adjusting for baseline BDI-II, STAI-total, and PCL-5 scores (Supplementary Fig. 7). No regions showed an association between WD changes and PHQ-15 score changes.

#### Cortical integration

Longitudinal integration changes were negatively associated with SOMS:CD score changes in the right anterior insula, orbitofrontal cortex, angular gyrus, lateral occipital cortex, and bilateral frontal pole and superior frontal gyri (Fig. 5B). Specifically, as integration in these regions decreased at follow-up compared to baseline, symptom scores improved. Findings largely held across all *post hoc* corrections aside from baseline BDI-II, STAI-total, and PCL-5 adjustments; additionally, the right insula finding did not hold when adjusting for CTQ-abuse and CTQ-neglect, or STAI-total change scores (Supplementary Fig. 5). For an additional inquiry into the discrete impact of the five domains of the CTQ, see Supplementary Fig. 4.

Longitudinal integration changes in the left lateral occipital pole and occipital fusiform gyrus were positively associated with PHQ-15 score changes (Supplementary Fig. 8), such that increased integration at follow-up compared to baseline was linked to physical symptom improvement. These findings did not hold for *post hoc* FND phenotype or CTQ adjustments but held for all other corrections (Supplementary Fig. 9).

#### *Post hoc* characterization of longitudinal integration-based network connectivity changes

**Figure 6:**
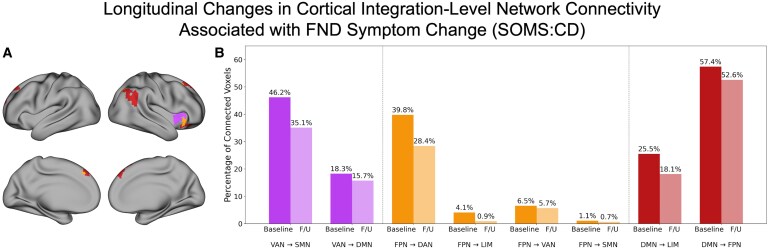
**Longitudinal changes in cortical integration-level network connectivity associated with functional neurological disorder (FND) symptom change (*N* = 28).** (**A**) Depicts voxels whose change in integration from baseline to follow-up was significantly associated with FND symptom change (ΔSOMS:CD scores), coloured according to their functional network assignment based on the Yeo *et al*. seven-network parcellation. Only the three networks containing the most significant voxels are displayed: ventral attention (VAN; magenta), frontoparietal (FPN; orange), and default mode network (DMN; red). For each of these networks, we extracted the average time series of all significant voxels for use in seed-to-voxel connectivity analyses, to then characterize the networks they were functionally integrating across. (**B**) Depicts a bar chart summarizing the between-network connectivity decreases for each seed. Darker bars reflect the per cent of connected voxels at baseline, while lighter bars reflect the per cent of connected voxels at follow-up. Only networks with decreased connections at follow-up compared to baseline are shown, given that a decrease in integration was associated with symptom improvement. For the VAN seed, connections with the SMN and DMN were relatively lower at follow-up compared to baseline. For the FPN seed, connections with LIM, VAN, and SMN were relatively lower at follow-up compared to baseline. For the DMN seed, connections with LIM and FPN were relatively lower at follow-up compared to baseline. F/U, follow-up; SMN, somatomotor network; DMN, default mode network; FPN, frontoparietal network; DAN, dorsal attention network; VAN, ventral attention network; LIM, limbic network. SOMS:CD, Screening for Somatoform Symptoms-7 Subscale for Conversion Disorder.

We again investigated which networks were functionally connected to the regions that showed significant associations between cortical integration changes (baseline versus follow-up rsFC) and SOMS:CD score changes via *post hoc* seed-to-voxel connectivity analyses. Most identified voxels were within VAN, FPN, and DMN, which were then used as seeds in this analysis. Compared to baseline rsFC observations, the VAN seed showed decreased connectivity at follow-up to SMN and DMN; the FPN seed showed decreased connectivity at follow-up to DAN, LIM, VAN, and SMN; the DMN seed showed decreased connectivity at follow-up to LIM and FPN (Fig. 6).

We also investigated which networks were functionally connected to the regions that showed significant associations between cortical integration changes and PHQ-15 score changes. Significant voxels fell within the VIS network, and showed increased connectivity with FPN, LIM, and DMN regions at follow-up compared to baseline (Supplementary Fig. 8).

#### Exploratory *post hoc* contextualization of longitudinal integration changes with HCs

**Figure 7:**
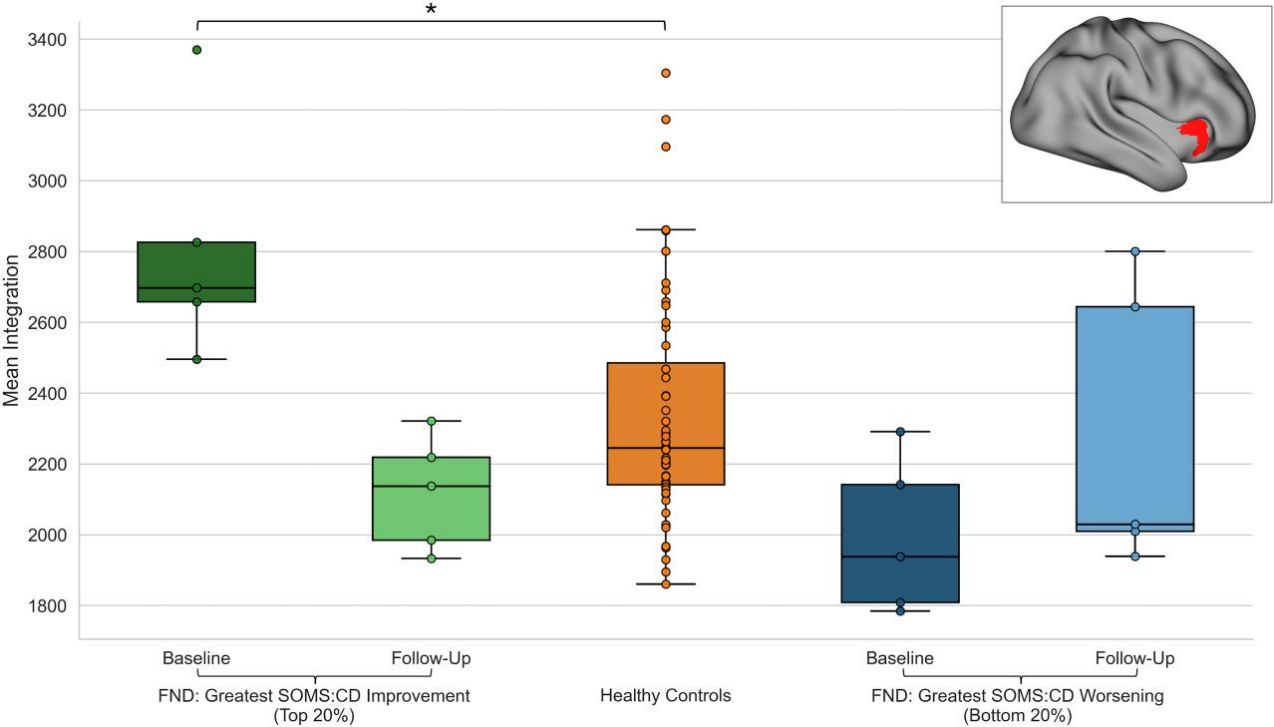
**Longitudinal changes in cortical integration-level network connectivity associated with functional neurological disorder (FND) symptom change (ΔSOMS:CD scores) in comparison to healthy controls.** Boxplots depict mean integration values for overlapping voxels (shown in red; *top right* overlay image) identified across baseline predictors and longitudinal change analyses. Plots are shown for the top 20% of FND participants with the greatest symptom improvement (*n* = 5; baseline: dark green, follow-up: light green), the bottom 20% of participants with the greatest symptom worsening (*n* = 5; baseline: dark blue, follow-up: light blue), and healthy control participants (*n* = 50; orange). Individual datapoints represent the mean integration value across overlapping voxels for a single participant at the indicated timepoint. Participants with the greatest symptom improvement exhibited decreases in mean integration values, whereas those with the greatest symptom worsening exhibited increases, with values predominantly falling within the healthy control range. Mean baseline integration values for the top five most improved participants were significantly higher than those of healthy controls (asterisk denotes *P* < 0.05, Mann–Whitney U-test). SOMS:CD, Screening for Somatoform Symptoms-7 Subscale for Conversion Disorder.

Mean integration values were extracted from overlapping voxels that emerged across baseline and longitudinal analyses (i.e. the right anterior insula). Individuals with the greatest improvement in SOMS:CD scores showed decreases in cortical integration from baseline to follow-up, whereas those with the greatest worsening showed modest increases, with integration values qualitatively falling predominantly within the upper and lower bounds of the HCs range (Fig. 7). Mann–Whitney U-tests revealed no significant differences in mean cortical integration between the *entire* FND cohort and HCs at baseline (*U* = 748*, P* = 0.59) and follow-up (*U* = 637, *P* = 0.50). However, the top 20% of individuals with the greatest improvement showed significantly higher baseline cortical integration relative to HCs (*U* = 30, *P* = 0.005). This difference was no longer evident at follow-up (*U* = 79, *P* = 0.16). In contrast, the bottom 20% of individuals with the greatest symptom worsening did not differ significantly from HCs at either baseline (*U* = 88, *P* = 0.24) or follow-up (*U* = 103, *P* = 0.45). Exploratory analyses stratifying by BDI-II or STAI-total change scores did not demonstrate the same pattern observed for SOMS:CD change scores (Supplementary Fig. 10).

#### Cortical segregation

No regions showed an association between segregation changes and changes in SOMS:CD or PHQ-15 scores from baseline to follow-up.

## Discussion

In this study, there were no group-level changes in psychometrically-measured core FND or non-core physical symptoms between baseline and follow-up; however, clinical trajectories varied considerably. Analyses using rsFC data and longitudinal psychometric scores revealed that heightened baseline connectivity in VAN, DMN, FPN, and DAN regions predicted greater symptom improvement at 6 months. Interestingly, longitudinal fMRI analyses showed that symptom improvement was associated with relative reductions in rsFC over time in many of these same networks. Overlap between predictors and mechanisms was observed for integrated (*between-network*) connectivity of the right anterior insula. Although mean cortical integration values for the entire FND cohort in this region fell within HCs ranges at both timepoints, stratified analyses showed that participants who improved the most displayed higher baseline integration relative to HCs, which normalized over time. The divergence between null group-level findings and individual differences in network organization underscores the importance of examining within-group variability in this population. Given that most of the cohort had FND-motor, it is noteworthy that findings remained significant adjusting for FND-seizure phenotype. However, several results, including the right anterior insula integration findings, attenuated when controlling for baseline affective symptoms and/or trauma burden—underscoring the biological and therapeutic relevance of these factors to longitudinal brain network reorganization in FND.^39^ Together, findings demonstrate the novel observations that hyperconnectivity at baseline, and its subsequent reduction over time, are linked to more favourable clinical trajectories in FND.

When examining baseline brain organization associations with change in SOMS:CD scores, individuals with relatively increased rsFC in VAN, FPN, and DMN regions had greater longitudinal improvement in core FND symptoms. Cortical integration analyses identified the right anterior insula and temporal pole as regions where higher between-network connectivity at baseline was related to greater improvement. The anterior insula, a core node of the VAN, has been implicated in FND pathophysiology (i.e. markers of symptom severity,^17^ impaired interoceptive processing^41^), including prior research linking improvement to increased left centromedial amygdala-right anterior insula connectivity.^17^  *Post hoc* seed-to-voxel analyses indicated that the baseline hyperconnectivity of the VAN insula seed was primarily driven by connections to the SMN, consistent with prior reports of heightened VAN-SMN coupling in FND.^22,42^ The insula cluster also overlapped with the FPN, which exhibited hyperconnectivity to both the DMN and VAN. Increased connectivity in other FPN regions (right middle frontal and precentral gyri) also predicted a favourable change in SOMS:CD scores across both whole-brain WD and cortical segregation analyses; the overlap across metrics suggests that the global centrality effects in these regions were driven by a greater number of within-FPN connections. This aligns with our prior research demonstrating increased FPN segregation in FND compared to psychiatric controls, albeit in a different subregion of the network.^22^ Baseline left cerebellum WD associations with change in SOMS:CD scores were also observed, spanning both motor and non-motor associative regions, implicating cerebellar systems as additional potential predictors of clinical improvement.^43^ The robustness of baseline rsFC findings varied across *post hoc* corrections for affective symptoms and trauma burden, suggesting that some observed associations may not be specific to FND symptom change alone. Overall, these results indicate that heightened connectivity at baseline—particularly between-network VAN, DMN, and FPN connections and within-FPN connections—is predictive of core FND symptom improvement.

Longitudinal mechanistic analyses (using fMRI and psychometric data collected across two timepoints) revealed that core FND symptom improvement was accompanied by functional connectivity decreases over time, consistent with the hypothesis of network reorganization accompanying symptom change. This association was particularly notable in the right anterior insula; *post hoc* analyses indicated that reduced right insula integration reflected decreases in VAN-SMN, VAN-DMN, and FPN-DAN connectivity. Notably, the right anterior insula integration finding remained significant after adjusting for a change in depressive symptoms but not after adjusting for a change in anxiety symptoms, suggesting that we could not fully determine whether this effect is specific to FND symptom change versus shared with anxiety-related processes. This finding also did not hold adjusting for baseline affective symptoms or trauma burden, and an additional cluster appeared (see Supplementary Fig. 7), highlighting the need to consider the role of these factors in future work. When contextualizing these findings against HCs, *post hoc* comparisons showed that right insula integration values in the *full* FND cohort did not differ from HCs at either timepoint, with values largely falling within the healthy range. In stratified exploratory *post hoc* analyses, however, the top 20% of individuals with the greatest symptom improvement demonstrated higher baseline integration relative to HCs. This difference was no longer evident at follow-up, suggesting a shift towards a normative range. Overall, the convergence of right anterior insula findings across baseline and longitudinal analyses underscores its potential as both a prognostic marker and as a mechanistic hub whose connectivity co-varies alongside symptom improvement.

Cortical integration analyses also identified that longitudinal connectivity decreases in the right angular gyrus/TPJ and bilateral superior frontal gyrus, regions of the DMN, were linked to SOMS:CD improvement; these effects mostly reflected reduced DMN-LIM and DMN-FPN coupling. These findings were robust across multiple *post hoc* corrections, yet attenuated when correcting for baseline depression, anxiety, and post-traumatic stress disorder (PTSD) symptoms. The right TPJ finding is notable given its implication in FND-related action-authorship perceptions and sensorimotor integration.^19,44^ Collectively, these results indicate that clinical improvement in core FND symptoms is accompanied by targeted reductions in VAN, FPN, and DMN connectivity. These findings may help guide emerging neuromodulation approaches for FND (e.g. transcranial magnetic stimulation), by identifying cortical regions that can serve as potential targets to modulate large-scale network interactions.^45^ Additionally, longitudinal WD associations were observed in the right cerebellum, spanning both motor and non-motor association regions, and remained significant across all *post hoc* adjustments for affective symptoms, trauma burden, and longitudinal changes in depression and anxiety symptoms. These observations highlight cerebellar circuitry as particularly stable potential mechanistic correlates of clinical improvement in FND. Although baseline predictor findings were left-lateralized and longitudinal effects were right-lateralized, these observations suggest that cerebellar circuits may contribute to both prognostic markers and dynamic mechanisms of symptom improvement in FND. Future work using whole-brain integration and segregation approaches that incorporate the cerebellum, as well as subcortical regions previously implicated in FND pathophysiology (e.g. basal ganglia, amygdala, hippocampus) will be important for refining and extending these network-level observations in FND.

For non-core physical symptoms measured by the PHQ-15, baseline analyses showed that higher cortical segregation in the left post-central and supramarginal gyri—both within the DAN—was linked to greater improvement, whereas lower segregation in these regions was associated with worsening. Mechanistically, improvement in PHQ-15 scores corresponded to increased integration of the VIS network from baseline to follow-up, with *post hoc* analyses showing strengthened connectivity to the FPN, DMN, and LIM networks. These results suggest that improvement in non-core physical symptoms may be supported by network patterns that are partly distinct from those underlying core FND symptoms, with an emphasis on sensory and attentional systems.^46,47^

Functional connectivity alterations in FND are increasingly being interpreted through the theoretical lens of predictive processing, which proposes that the brain draws on past experiences to actively construct predictions about incoming sensory input, rather than simply reacting.^48-53^ The DMN, FPN, and salience network (closely related to the VAN in the Yeo *et al*. parcellation used in this paper; see limitation section for additional qualification) have been hypothesized to be involved in aspects of predictive processing, providing one potential framework for considering network-level alterations.^54-56^ While speculative, the present findings, in which heightened baseline connectivity across these networks predicted improvement and symptom improvement was associated with reductions in these same connections, may be broadly consistent with changes in maladaptive predictive processes in FND.

This study has limitations. Several factors may have contributed to the lack of group-level differences in clinical outcomes, including the heterogeneity of FND presentations and clinical trajectories, individualized non-standardized treatment exposures inherent to the naturalistic design, incomplete longitudinal follow-up data, and relatively broad enrolment criteria compared with controlled clinical trials in FND.^57-59^ The naturalistic design increases ecological validity but introduces heterogeneity in treatment exposures. Additionally, the observational design precludes direct causal inferences. However, as the goal of this study was to examine neural features associated with individual differences in symptom change, rather than to evaluate a specific intervention, variability in treatment is inherent to the design. Prior work from our group identified positive associations between the number of treatment sessions and FND clinical outcomes,^35,36^ underscoring the importance of systematically capturing treatment details in future larger-scale research. Symptom trajectories were assessed via self-report, which is the favoured approach for evaluating symptom change in FND based on international recommendations^60^; however, the inclusion of clinician-rated or objective measures of symptom change will be an important future addition. The absence of a control group of FND participants who did not receive treatment also limits our ability to estimate the natural course of symptom change. Future studies might also include prospective enrolment of and longitudinal follow-up of a psychiatric comparator group (e.g. individuals with major depressive disorder, anxiety disorders, PTSD) to clarify disorder-specific versus shared neural mechanisms of symptom change. The specific functional connectivity patterns observed may also depend in part on the resting-state network parcellation employed.^61^ Future studies using alternative and more granular parcellation schemes—including those distinguishing between the VAN, salience, and cingulo-opercular action mode networks^62-65^—may further refine understanding of the implicated networks underlying symptom change in FND. Further, several findings attenuated when accounting for baseline affective symptoms or trauma burden, indicating that they are likely components of the same complex system rather than independent factors, and should therefore be modelled as such in future research. PTSD symptoms (PCL-5) were not assessed at follow-up, precluding adjustment for longitudinal changes in trauma-related symptoms. There is also a need to further consider the impact of types of childhood maltreatment, the developmental stage at which an individual experiences adversity, and cumulative trauma burden. Given that a subset of initially enrolled participants were lost to follow-up, replication in larger FND cohorts with more controlled treatment protocols and multimodal physiological assessments will be critical for refining and validating these network-level observations. Overall, more research is needed to fully determine if the study findings have direct mechanistic relevance or if they potentially reflect less specific features such as compensatory processes.

In conclusion, this fMRI study identified rsFC features linked to symptom change in FND. Greater baseline hyperconnectivity—particularly involving the integrated connectivity of the right anterior insula—predicted improvement, while recovery was characterized by reductions in these same connections. These findings highlight the potential of large-scale network interactions to serve as prognostic markers and elucidate mechanistic insights that set the stage for the development of novel, biologically informed interventions.

## Supplementary Material

fcag290_Supplementary_Data

## Data Availability

For qualified researchers, de-identified data pertaining only to study results can be made available following local IRB approval upon reasonable request. Analysis code can be found in the following GitHub repository: https://github.com/FND-RG-Lab/NetworkIntegrationSegregation.

## References

[1] Hallett M, Aybek S, Dworetzky BA, McWhirter L, Staab JP, Stone J. Functional neurological disorder: New subtypes and shared mechanisms. Lancet Neurol. 2022;21:537–550. 35430029 10.1016/S1474-4422(21)00422-1 PMC9107510

[2] Jones B, Reuber M, Norman P. Correlates of health-related quality of life in adults with psychogenic nonepileptic seizures: A systematic review. Epilepsia. 2016;57:171–181. 26701628 10.1111/epi.13268

[3] Stephen CD, Fung V, Perez DL, Espay AJ. Comparison of inpatient and emergency department costs to research funding for functional neurologic disorder: An economic analysis. Neurology. 2025;104:e213445. 39999398 10.1212/WNL.0000000000213445 PMC11863780

[4] Matin N, Young SS, Williams B, et al Neuropsychiatric associations with gender, illness duration, work disability, and motor subtype in a U.S. functional neurological disorders clinic population. J Neuropsychiatry Clin Neurosci. 2017;29:375–382. 28449634 10.1176/appi.neuropsych.16110302

[5] Tinazzi M, Morgante F, Marcuzzo E, et al Clinical correlates of functional motor disorders: An Italian multicenter study. Mov Disord Clin Pract. 2020;7:920–929. 33163563 10.1002/mdc3.13077 PMC7604660

[6] McKenzie PS, Oto M, Graham CD, Duncan R. Do patients whose psychogenic non-epileptic seizures resolve, “replace” them with other medically unexplained symptoms? Medically unexplained symptoms arising after a diagnosis of psychogenic non-epileptic seizures. J Neurol Neurosurg Psychiatry. 2011;82:967–969. 21421771 10.1136/jnnp.2010.231886

[7] Perez DL, Finkelstein S, Adams C, Saxena A. Toward a precision medicine approach to the outpatient assessment and treatment of functional neurological disorder. Neurol Clin. 2023;41:681–693. 37775198 10.1016/j.ncl.2023.02.006

[8] Sharpe M, Walker J, Williams C, et al Guided self-help for functional (psychogenic) symptoms: A randomized controlled efficacy trial. Neurology. 2011;77:564–572. 21795652 10.1212/WNL.0b013e318228c0c7 PMC3149156

[9] Nielsen G, Stone J, Lee TC, et al Specialist physiotherapy for functional motor disorder in England and Scotland (Physio4FMD): A pragmatic, multicentre, phase 3 randomised controlled trial. Lancet Neurol. 2024;23:675–686. 38768621 10.1016/S1474-4422(24)00135-2

[10] Goldstein LH, Robinson EJ, Mellers JDC, et al Cognitive behavioural therapy for adults with dissociative seizures (CODES): A pragmatic, multicentre, randomised controlled trial. Lancet Psychiatry. 2020;7:491–505. 32445688 10.1016/S2215-0366(20)30128-0 PMC7242906

[11] Gelauff JM, Carson A, Ludwig L, Tijssen MAJ, Stone J. The prognosis of functional limb weakness: A 14-year case-control study. Brain. 2019;142:2137–2148. 31167232 10.1093/brain/awz138

[12] Nielsen G, Lee TC, Marston L, et al Which factors predict outcome from specialist physiotherapy for functional motor disorder? Prognostic modelling of the Physio4FMD intervention. J Psychosom Res. 2025;190:112056. 39952010 10.1016/j.jpsychores.2025.112056

[13] Goldstein LH, Robinson EJ, Chalder T, et al Moderators of cognitive behavioural therapy treatment effects and predictors of outcome in the CODES randomised controlled trial for adults with dissociative seizures. J Psychosom Res. 2022;158:110921. 35617911 10.1016/j.jpsychores.2022.110921

[14] Sekine ER, Kanaan RA, McMillan J, Oxford S, Iles RA. Biopsychosocial prognostic indicators in functional neurological disorder: A systematic review. J Psychosom Res. 2025;195:112201. 40609303 10.1016/j.jpsychores.2025.112201

[15] Perez DL, Nicholson TR, Asadi-Pooya AA, et al Neuroimaging in functional neurological disorder: State of the field and research agenda. Neuroimage Clin. 2021;30:102623. 34215138 10.1016/j.nicl.2021.102623 PMC8111317

[16] Baek K, Doñamayor N, Morris LS, et al Impaired awareness of motor intention in functional neurological disorder: Implications for voluntary and functional movement. Psychol Med. 2017;47:1624–1636. 28183377 10.1017/S0033291717000071 PMC5964459

[17] Diez I, Ortiz-Terán L, Williams B, et al Corticolimbic fast-tracking: Enhanced multimodal integration in functional neurological disorder. J Neurol Neurosurg Psychiatry. 2019;90:929–938. 30850473 10.1136/jnnp-2018-319657 PMC6625895

[18] Li R, Li Y, An D, Gong Q, Zhou D, Chen H. Altered regional activity and inter-regional functional connectivity in psychogenic non-epileptic seizures. Sci Rep. 2015;5:11635. 26109123 10.1038/srep11635 PMC4480007

[19] Maurer CW, LaFaver K, Ameli R, Epstein SA, Hallett M, Horovitz SG. Impaired self-agency in functional movement disorders. Neurology. 2016;87:564–570. 27385746 10.1212/WNL.0000000000002940 PMC4977370

[20] van der Kruijs SJM, Bodde NMG, Vaessen MJ, et al Functional connectivity of dissociation in patients with psychogenic non-epileptic seizures. J Neurol Neurosurg Psychiatry. 2012;83:239–247. 22056967 10.1136/jnnp-2011-300776

[21] Szaflarski JP, LaFrance WC, Nenert R, et al Neurobehavioral therapy in functional seizures: Investigation of mechanism of action with resting-state functional magnetic resonance imaging. Epilepsia. 2025;66:2881–2893. 40183564 10.1111/epi.18401 PMC12353879

[22] Westlin C, Guthrie AJ, Bleier C, et al Delineating network integration and segregation in the pathophysiology of functional neurological disorder. Brain Commun. 2025;7:fcaf195. 40433115 10.1093/braincomms/fcaf195 PMC12107243

[23] Conejero I, Collombier L, Lopez-Castroman J, et al Association between brain metabolism and clinical course of motor functional neurological disorders. Brain. 2022;145:3264–3273. 35445242 10.1093/brain/awac146

[24] Espay AJ, Ries S, Maloney T, et al Clinical and neural responses to cognitive behavioral therapy for functional tremor. Neurology. 2019;93:e1787–e1798. 31586023 10.1212/WNL.0000000000008442 PMC6946484

[25] Faul L, Knight LK, Espay AJ, Depue BE, LaFaver K. Neural activity in functional movement disorders after inpatient rehabilitation. Psychiatry Res Neuroimaging. 2020;303:111125. 32585576 10.1016/j.pscychresns.2020.111125

[26] Schneider A, Weber S, Wyss A, Loukas S, Aybek S. BOLD signal variability as potential new biomarker of functional neurological disorders. Neuroimage Clin. 2024;43:103625. 38833899 10.1016/j.nicl.2024.103625 PMC11179625

[27] LaFrance WC, Baker GA, Duncan R, Goldstein LH, Reuber M. Minimum requirements for the diagnosis of psychogenic nonepileptic seizures: A staged approach: A report from the international league against epilepsy nonepileptic seizures task force. Epilepsia. 2013;54:2005–2018. 24111933 10.1111/epi.12356

[28] Westlin C, Guthrie AJ, Paredes-Echeverri S, et al Machine learning classification of functional neurological disorder using structural brain MRI features. J Neurol Neurosurg Psychiatry. 2025;96:249–257. 39033019 10.1136/jnnp-2024-333499 PMC11743827

[29] Westlin C, Guthrie A, Bleier C, et al Functional connectivity gradients reveal altered hierarchical cortical organization in functional neurological disorder. Biol Psychiatry Cogn Neurosci Neuroimaging. 2026;11:597–609. 41167519 10.1016/j.bpsc.2025.10.010 PMC12649792

[30] Myers L, Sarudiansky M, Korman G, Baslet G. Using evidence-based psychotherapy to tailor treatment for patients with functional neurological disorders. Epilepsy Behav Rep. 2021;16:100478. 34693243 10.1016/j.ebr.2021.100478 PMC8515382

[31] Baker J, Barnett C, Cavalli L, et al Management of functional communication, swallowing, cough and related disorders: Consensus recommendations for speech and language therapy. J Neurol Neurosurg Psychiatry. 2021;92:1112–1125. 34210802 10.1136/jnnp-2021-326767

[32] Nicholson C, Edwards MJ, Carson AJ, et al Occupational therapy consensus recommendations for functional neurological disorder. J Neurol Neurosurg Psychiatry. 2020;91:1037–1045. 32732388 10.1136/jnnp-2019-322281

[33] Nielsen G, Stone J, Matthews A, et al Physiotherapy for functional motor disorders: A consensus recommendation. J Neurol Neurosurg Psychiatry. 2015;86:1113–1119. 25433033 10.1136/jnnp-2014-309255 PMC4602268

[34] LaFrance Jr WC, Baird GL, Barry JJ, et al Multicenter pilot treatment trial for psychogenic nonepileptic seizures: A randomized clinical trial. JAMA Psychiatry. 2014;71:997–1005. 24989152 10.1001/jamapsychiatry.2014.817

[35] Bleier C, Godena E, Millstein D, et al Predictors of skills-based psychotherapy outcomes for functional neurological disorder: A retrospective cohort study. J Neuropsychiatry Clin Neurosci. 2026;38(2):194–200. 41039862 10.1176/appi.neuropsych.20250011

[36] Maggio JB, Ospina JP, Callahan J, Hunt AL, Stephen CD, Perez DL. Outpatient physical therapy for functional neurological disorder: A preliminary feasibility and naturalistic outcome study in a U.S. cohort. J Neuropsychiatry Clin Neurosci. 2020;32:85–89. 31564236 10.1176/appi.neuropsych.19030068

[37] Rief W, Hiller W. A new approach to the assessment of the treatment effects of somatoform disorders. Psychosomatics. 2003;44:492–498. 14597684 10.1176/appi.psy.44.6.492

[38] Kroenke K, Spitzer RL, Williams JBW. The PHQ-15: Validity of a new measure for evaluating the severity of somatic symptoms. Psychosom Med. 2002;64:258–266. 11914441 10.1097/00006842-200203000-00008

[39] Diez I, Larson AG, Nakhate V, et al Early-life trauma endophenotypes and brain circuit–gene expression relationships in functional neurological (conversion) disorder. Mol Psychiatry. 2021;26:3817–3828. 32051548 10.1038/s41380-020-0665-0 PMC7423688

[40] Yeo BTT, Krienen FM, Sepulcre J, et al The organization of the human cerebral cortex estimated by intrinsic functional connectivity. J Neurophysiol. 2011;106:1125–1165. 21653723 10.1152/jn.00338.2011 PMC3174820

[41] Sojka P, Serranová T, Khalsa SS, Perez DL, Diez I. Altered neural processing of interoception in patients with functional neurological disorder: A task-based fMRI study. J Neuropsychiatry Clin Neurosci. 2025;37(2):149–159. 39558709 10.1176/appi.neuropsych.20240070

[42] Voon V, Brezing C, Gallea C, et al Emotional stimuli and motor conversion disorder. Brain. 2010;133:1526–1536. 20371508 10.1093/brain/awq054 PMC2859149

[43] Stoodley CJ, Schmahmann JD. Functional topography of the human cerebellum. Handb Clin Neurol. 2018;154:59–70. 29903452 10.1016/B978-0-444-63956-1.00004-7

[44] Voon V, Gallea C, Hattori N, Bruno M, Ekanayake V, Hallett M. The involuntary nature of conversion disorder. Neurology. 2010;74:223–228. 20083798 10.1212/WNL.0b013e3181ca00e9 PMC2809033

[45] Oriuwa C, Mollica A, Feinstein A, et al Neuromodulation for the treatment of functional neurological disorder and somatic symptom disorder: A systematic review. J Neurol Neurosurg Psychiatry. 2022;93:280–290. 35115389 10.1136/jnnp-2021-327025

[46] Věchetová G, Nikolai T, Slovák M, et al Attention impairment in motor functional neurological disorders: A neuropsychological study. J Neurol. 2022;269:5981–5990. 35842882 10.1007/s00415-022-11211-x

[47] Huys A-CML, Haggard P, Bhatia KP, Edwards MJ. Misdirected attentional focus in functional tremor. Brain. 2021;144:3436–3450. 34145898 10.1093/brain/awab230 PMC8677517

[48] Edwards MJ, Adams RA, Brown H, Pareés I, Friston KJ. A Bayesian account of ‘hysteria’. Brain. 2012;135:3495–3512. 22641838 10.1093/brain/aws129 PMC3501967

[49] Jungilligens J, Paredes-Echeverri S, Popkirov S, Barrett LF, Perez DL. A new science of emotion: Implications for functional neurological disorder. Brain. 2022;145:2648–2663. 35653495 10.1093/brain/awac204 PMC9905015

[50] Sadnicka A, Daum C, Meppelink A-M, Manohar S, Edwards M. Reduced drift rate: A biomarker of impaired information processing in functional movement disorders. Brain. 2020;143:674–683. 31865371 10.1093/brain/awz387

[51] Lin D, Castro P, Edwards A, et al Dissociated motor learning and de-adaptation in patients with functional gait disorders. Brain. 2020;143:2594–2606. 32779724 10.1093/brain/awaa190

[52] Pareés I, Kassavetis P, Saifee TA, et al “Jumping to conclusions” bias in functional movement disorders. J Neurol Neurosurg Psychiatry. 2012;83:460–463. 22338028 10.1136/jnnp-2011-300982

[53] Pareés I, Saifee TA, Kassavetis P, et al Believing is perceiving: Mismatch between self-report and actigraphy in psychogenic tremor. Brain. 2012;135:117–123. 22075068 10.1093/brain/awr292

[54] Barrett LF . The theory of constructed emotion: An active inference account of interoception and categorization. Soc Cogn Affect Neurosci. 2017;12:1833. 28472391 10.1093/scan/nsx060 PMC5691871

[55] Kleckner IR, Zhang J, Touroutoglou A, et al Evidence for a large-scale brain system supporting allostasis and interoception in humans. Nat Hum Behav. 2017;1:0069. 28983518 10.1038/s41562-017-0069 PMC5624222

[56] Satpute AB, Lindquist KA. The default mode network’s role in discrete emotion. Trends Cogn Sci. 2019;23:851–864. 31427147 10.1016/j.tics.2019.07.003 PMC7281778

[57] Gilmour GS, Langer LK, Bhatt H, MacGillivray L, Lidstone SC. Factors influencing triage to rehabilitation in functional movement disorder. Mov Disord Clin Pract. 2024;11:515–525. 38385766 10.1002/mdc3.14007 PMC11078488

[58] Jalilianhasanpour R, Ospina JP, Williams B, et al Secure attachment and depression predict 6-month outcome in motor functional neurological disorders: A prospective pilot study. Psychosomatics. 2019;60:365–375. 30342702 10.1016/j.psym.2018.08.004

[59] Issak S, Nielsen G, Fini NA, Kanaan RA, Williams G. Longitudinal gait changes in functional neurological disorder: A 12-month prospective study. J Psychosom Res. 2025;199:112408. 41172707 10.1016/j.jpsychores.2025.112408

[60] Pick S, Anderson DG, Asadi-Pooya AA, et al Outcome measurement in functional neurological disorder: A systematic review and recommendations. J Neurol Neurosurg Psychiatry. 2020;91:638–649. 32111637 10.1136/jnnp-2019-322180 PMC7279198

[61] Uddin LQ, Yeo BTT, Spreng RN. Towards a universal taxonomy of macro-scale functional human brain networks. Brain Topogr. 2019;32:926–942. 31707621 10.1007/s10548-019-00744-6 PMC7325607

[62] Seeley WW, Menon V, Schatzberg AF, et al Dissociable intrinsic connectivity networks for salience processing and executive control. J Neurosci. 2007;27(9):2349–2356. 17329432 10.1523/JNEUROSCI.5587-06.2007 PMC2680293

[63] Dosenbach NU, Fair DA, Miezin FM, et al Distinct brain networks for adaptive and stable task control in humans. Proc Natl Acad Sci U S A. 2007;104(26):11073–11078. 17576922 10.1073/pnas.0704320104 PMC1904171

[64] Dosenbach NUF, Raichle ME, Gordon EM. The brain’s action-mode network. Nat Rev Neurosci. 2025;26(3):158–168. 39743556 10.1038/s41583-024-00895-x

[65] Dworetsky A, Seitzman BA, Adeyemo B, et al Probabilistic mapping of human functional brain networks identifies regions of high group consensus. Neuroimage. 2021;237:118164. 34000397 10.1016/j.neuroimage.2021.118164 PMC8296467

