# Supplementary material for: Functional connectivity predictors and mechanisms of symptom change in functional neurological disorder
Source: Brain Commun. 2026 Jul 22;8(4):fcag290. doi: 10.1093/braincomms/fcag290 (PMC13435623; doi:10.1093/braincomms/fcag290)
Supplement: Supplementary file 1 [file fcag290_supplementary_data.docx]

**SUPPLEMENTARY MATERIALS**

**Laboratory Designed Follow-up Questions**

1). In the past 6 months, have you participated in physical therapy?

Yes	No

1.a). If yes, how many sessions did you participate in over the past 6 months?

0-5

6-10

11-15

16-20

>20

2). In the past 6 months, have you participated in occupational therapy?

Yes	No

2.a). If yes, how many sessions did you participate in over the past 6 months?

0-5

6-10

11-15

16-20

>20

3). In the past 6 months, have you participated in speech and language therapy?

Yes	No

3.a). If yes, how many sessions did you participate in over the past 6 months?

0-5

6-10

11-15

16-20

>20

4). In the past 6 months, have you participated in individual psychotherapy?

Yes	No

4.a). If yes, how many sessions did you participate in over the past 6 months?

0-5

6-10

11-15

16-20

>20

4.b). If yes, are you participating in cognitive behavioral therapy?

Yes	No

5). In the past 6 months, have you participated in group psychotherapy?

Yes	No

5.a). If yes, how many sessions did you participate in over the past 6 months?

0-5

6-10

11-15

16-20

>20

6). In the last 6 months, did you use in any capacity the workbook "Overcoming Functional Neurological Symptoms: A Five Area Approach"?

Yes	No

7). In the last 6 months, did you use in any capacity the workbook "Taking Control of Your Seizures: Workbook (Treatments That Work)"?

Yes	No

8). Select below the option that best describes your functional neurological disorder treatment.

- All treatment within the Mass General Brigham system

- Some community and some Mass General Brigham system

- No treatment received within the Mass General Brigham system

9). Are you currently (Check all that apply):

- Unemployed

- Student

- Homemaker

- Working part time

- Working full time

- Retired

- On disability

- Applying for disability

- Other

10). Are you currently (Check all that apply):

- Single

- In a relationship

- Engaged

- Married

- Divorced

- Widowed

- Other

**SUPPLEMENTARY TABLES**

**Supplementary Table 1. Demographic characteristics of participants with functional neurological disorder (FND).**

| **FND Subject** | **FND**  **Phenotype** | **Phenotype Descriptions** | **Current (Baseline) SCID-DSM-5 Diagnoses** | **Past SCID-DSM-5 Diagnoses** | **Baseline**  **Psychotropic Medications** | **Follow-up**  **Psychotropic Medications** |
| --- | --- | --- | --- | --- | --- | --- |
| 1 | FND-Motor | clinically-established functional tremor & functional gait | MDE, SAD | MDE, PD+AG | FLX, CLP | DLX, GBP, CLP |
| 2 | FND-Motor | clinically-established functional gait & functional speech | PTSD, Eating Disorder | MDE | QTP, ECP, CLP, HDZ, PZN | VNF, QTP, HDZ, PZN, CLP |
| 3 | FND-Seizure | documented functional seizures | DEP NOS | MDE, AG | NRT, LTG | LTG, PZN, NRT, HDZ |
| 4 | FND-Motor** | clinically-established functional tremor | GAD, IAD | MDE, PTSD | DLX, LRZ | LRZ, DLX |
| 5* | FND-Motor | clinically-established functional jerky movements | GAD | Eating Disorder, AUD, SUD | ECP, PGB, AMT, APM / DXAM | ECP |
| 6 | FND-Motor | clinically-established functional tremor | ANX NOS | - | GBP | GBP |
| 7* | FND-Seizure | documented functional seizures | - | DEP NOS, SAD, ANX NOS | LRZ | LRZ |
| 8 | FND-Seizure | probable functional seizures | ANX NOS | PTSD, GAD, MDE | SERT, TZD, PZN | SERT, TZD |
| 9 | FND-Motor | clinically-established functional jerky movements | GAD | DEP NOS | SERT | SERT |
| 10 | FND-Motor | clinically-established functional limb weakness (left arm) & functional speech | GAD, SSD | MDE, PTSD | CBD | - |
| 11 | FND-Seizure | probable functional seizures | - | ANX NOS | - | - |
| 12 | FND-Seizure, FND-Motor | clinically-established functional tremor, probable functional seizures | - | MDE | AMT | AMT |
| 13 | FND-Motor** | clinically-established functional tremor & functional speech | PTSD, GAD, PD+AG, MDE | - | DLX, BUP, LRZ | DLX, BUP, LRZ |
| 14 | FND-Motor | clinically-established functional gait & functional speech | AG, ADHD | - | APZ, CBD | APZ |
| 15 | FND-Seizure, FND-Motor | documented functional seizures; clinically-established functional gait & functional speech | ANX NOS | PTSD, PD+AG | PGB, CBD | PGB |
| 16 | FND-Motor | clinically-established functional gait, functional tremor, functional limb weakness (right arm/leg), & functional speech | GAD, PD+AG | PTSD, MDE | TPM | TPM |
| 17 | FND-Motor** | clinically-established functional gait | ANX NOS, SSD | GAD, MDE | SERT, LRZ, GBP | SERT, LRZ, GBP |
| 18 | FND-Motor | clinically-established functional tremor | - | PTSD, BPAD-II (with MDE) | LTM, QTP, LRZ | LTM, QTP, LRZ |
| 19 | FND-Motor | clinically-established functional limb weakness (legs) & functional speech | - | DEP NOS, Specific Phobia | - | - |
| 20 | FND-Motor | clinically-established functional tremor, functional dystonia & functional gait | PD+AG, SSD | GAD, DEP NOS | - | ATX |
| 21 | FND-Motor | clinically-established functional facial spasms/tics & functional speech | GAD, PD+AG, ADHD, PTSD | MDE | CLN, LDA, APR, SERT, LRZ | CLN, LDA, SERT |
| 22 | FND-Motor | clinically-established functional gait & functional speech | - | DEP NOS, ANX NOS | GBP | GBP |
| 23 | FND-Motor | clinically-established functional limb weakness (left leg) | ANX NOS | MDE, ANX NOS | GBP, HDZ, ECP | ECP, DLX, HDZ |
| 24 | FND-Seizure, FND-Motor | probable functional seizures; clinically-established functional limb weakness (bilateral leg), functional tremor, & functional gait | SSD, AG, DYS | MDE, PD-AG, GAD, PTSD, Eating Disorder | AMT, DLX | AMT, DLX |
| 25 | FND-Motor | clinically-established functional dystonia & functional speech | GAD | PTSD | - | - |
| 26 | FND-Motor | clinically-established functional limb weakness (left arm & leg), functional tremor, & functional jerks | - | PTSD | - | - |
| 27 | FND-Motor | clinically-established functional tremor | PTSD, MDE, Anxiety NOS | - | MIR, CLP | MIR, CLP |
| 28 | FND-Seizure, FND-Motor | documented functional seizures; clinically-established functional jerks/tics, & functional speech | PD+AG, DYS, GAD, SSD | OCD | DLX, GBP, LRZ | AMA, VNF, HDZ |
| 29* | FND-Seizure | documented functional seizures | AG, SAD, OCD, SSD | - | HDZ, DZP | HDZ, DZP, SERT |
| 30* | FND-Motor | clinically-established functional tremor, & functional gait | Anxiety NOS | - | HDZ | - |
| 31 | FND-Seizure, FND-Motor | documented functional seizures; clinically-established functional gait, functional limb weakness (left arm and leg), & functional speech | PTSD, MDE, PD+AG, GAD, SSD | MDE, Eating Disorder, AUD, SUD | BUP, GBP, TZD, HDZ | BUP, GBP, HDZ, TZD |
| 32 | FND-Motor | clinically-established functional limb weakness (left arm and leg) & functional gait | - | MDE, SAD, PTSD, Eating Disorder | CTP | CTP |

*Indicates subjects who did not have a scan at the 6-month follow-up session. **Indicates subject also had concurrent functional somatosensory loss (e.g., non-dermatomal somatosensory deficits). FND-Motor, Functional Motor Disorder; FND-Seizure, Functional Seizures; SCID-DSM-5, Structured Clinical Interview for DSM 5^th^ Edition [note subject 1 received a SCID-I for DSM-IV]; ADHD, Attention-Deficit/Hyperactivity Disorder; AG, Agoraphobia; ANX, Anxiety; AUD, Alcohol Use Disorder; BPAD, Bipolar Affective Disorder; DEP, Depression; DYS, Dysthymia; GAD, Generalized Anxiety Disorder; IAD, Illness Anxiety Disorder; MDE, Major Depressive Episode; NOS, not otherwise specified; OCD, Obsessive Compulsive Disorder; PD+AG, Panic Disorder with Agoraphobia; PD-AG, Panic Disorder without Agoraphobia; PTSD, Post-Traumatic Stress Disorder; SAD, Social Anxiety Disorder; SSD, Somatic Symptom Disorder; SUD, Substance Use Disorder; AMA, Amantadine; AMT, Amitriptyline; APM, Amphetamine; APR, Aripiprazole; APZ, Alprazolam; ATX, Atomoxetine; BUP, Bupropion; CBD, Cannabidiol; CLN, Clonidine; CLP, Clonazepam; CTP, Citalopram; DLX, Duloxetine; DXAM, Dextroamphetamine; DZP, Diazepam; ECP, Escitalopram; FLX, Fluoxetine; GBP, Gabapentin; HDZ, Hydroxyzine; LDA, Lisdexamfetamine; LTM, Lithium; LTG, Lamotrigine; LRZ, Lorazepam; MIR, Mirtazapine; NRT, Nortriptyline; PGB, Pregabalin; PZN, Prazosin; QTP, Quetiapine; SERT, Sertraline; TPM, Topiramate; TZD, Trazodone; VNF, Venlafaxine.

**Supplementary Table 2. Baseline characteristics of participants who completed vs. did not complete 6-month follow-up sessions.**

|  | **Participants who completed follow-up (n=32)** Mean ± SD or n (%) | **Participants lost to follow-up (n=20)**  Mean ± SD or n (%) | **Test statistic** | **Uncorrected**  **p-value** | **FDR corrected p-value** |
| --- | --- | --- | --- | --- | --- |
| **Age at consent (yrs.)** | 42.4 ± 13.3; range: 22.4-72.1; median: 44.0 | 37.1 ± 13.8; range: 19.6-65.8; median: 33.9 | 1.24^a^ | 0.17 | 0.44 |
| **Biological sex** | 28F 4M | 17F 3M | 0.07^b^ | 0.80 | 0.80 |
| **Illness duration (yrs.)** | 3.7 ± 4.2; range: 0.1-16.1; median: 2.1 | 2.3 ± 1.4; range: 0.5-5.4; median: 1.8 | 325.0^c^ | 0.46 | 0.66 |
| **Race (white)** | 28 (87.5%) | 17 (85.0%) | 0.07^b^ | 0.79 | 0.80 |
| **Married** | 15 (46.9%) | 8 (40.0%) | 0.24^b^ | 0.63 | 0.74 |
| **College graduate** | 22 (68.8%) | 10 (50.0%) | 1.00^b^ | 0.32 | 0.57 |
| **Employed (or student full time)** | 12 (37.5%) | 5 (25.0%) | 0.87^b^ | 0.35 | 0.57 |
| **On medical disability** | 10 (31.3%) | 8 (40.0%) | 0.32^b^ | 0.32 | 0.57 |
| **Functional motor disorder** | 27 (84.4%) | 11 (55.0%) | 3.90^b^ | 0.05 | 0.21 |
| **Functional seizures** | 10 (31.3%) | 8 (40.0%) | 0.42^b^ | 0.52 | 0.67 |
| **Taking SSRI/SNRI at baseline** | 13 (40.6%) | 12 (60.0%) | 2.93^b^ | 0.09 | 0.28 |
| **Baseline SOMS:CD** | 6.6 ± 6.3, range: 0-21; median: 5 | 12.2 ± 8.7, range: 0-28; median: 11 | 234.5^c^ | 0.03 | 0.21 |
| **Baseline STAI-state** | 38.3 ± 11.6, range: 20-68; median: 35 | 42.6 ± 8.9, range: 31-65; median: 39 | 237.5^c^ | 0.05 | 0.21 |

Participants were classified as lost to research follow-up if they did not complete the follow-up session after at least two contact attempts. ^a^ Test statistic corresponds to t-test. ^b^ Test statistic corresponds to chi-square test. ^c^ Test statistic corresponds to Mann-Whitney U test. SSRI, Selective Serotonin Reuptake Inhibitor; SNRI, Serotonin and Norepinephrine Reuptake Inhibitor, SOMS:CD Screening for Somatoform Symptoms-7 Subscale for Conversion Disorder; STAI-state, Spielberger State-Trait Anxiety Inventory-State subscale.

**Supplementary Table 3.** **Responses to follow-up questionnaire assessing therapy participation, treatment setting, and sociodemographic factors.**

| **Laboratory Designed Questions** | **Responses (n=32)** |
| --- | --- |
| 1. Physical therapy | Yes: 17, No: 15 |
| 1.a. Physical therapy sessions | (0-5): 4  (6-10): 3  (11-15): 1  (16-20): 2  (>20): 7 |
| 2. Occupational therapy^a^ | Yes: 16^a^, No: 16 |
| 2.a. Occupational therapy sessions^a^ | (0-5): 6  (6-10): 6  (11-15): 1  (16-20): 0  (>20): 3 |
| 3. Speech and language therapy^a^ | Yes: 10, No: 22 |
| 3.a. Speech and language therapy sessions^a^ | (0-5): 7  (6-10): 3  (11-15): 0  (16-20): 0  (>20): 0 |
| 4. Individual psychotherapy^a^ | Yes: 29, No: 3 |
| 4.a. Individual psychotherapy sessions^a^ | (0-5): 8  (6-10): 6  (11-15): 5  (16-20): 10  (>20): 0 |
| 4.b. Cognitive-behavioral therapy^a^ | Yes: 20, No: 9 |
| 5. Group psychotherapy^a^ | Yes: 5, No: 27 |
| 5.a. Group psychotherapy sessions^a^ | (0-5): 0  (6-10): 4  (11-15): 0  (16-20): 1  (>20): 0 |
| 6. Used workbook *“Overcoming Functional Neurological Symptoms: A Five Area Approach”*? | Yes: 17, No: 15 |
| 7. Used workbook *“Taking Control of Your Seizures: Workbook (Treatments That Work)”*? | Yes: 5, No: 27 |
| 8. FND treatment setting | - All treatment within the Mass General Brigham system: 11  - Some community and some Mass General Brigham system: 16  - No treatment received within the Mass General Brigham system: 5 |
| 9. Employment status (all that apply) | Unemployed 5  Student 3  Homemaker 2  Working part time 10  Working full time 14  Retired 0  On disability 7  Applying for disability 5  Other 1 |
| 10. Relationship status (all that apply) | Single 7  In a relationship 7  Engaged 1  Married 15  Divorced 4  Widowed 2  Other 0 |

^a^ Two participants self-reported not receiving psychotherapy in the 6 months prior to the follow-up questionnaire. However, cross-reference with available medical records revealed that they had both participated in individual psychotherapy and one of them had also participated in group psychotherapy during that period. In total, 30 of 32 individuals received some form of psychotherapy (individual and/or group) in between their baseline and follow-up assessments; 21 of 32 received a component of cognitive behavioral therapy (individual and/or group) in their psychotherapy. Similarly, two participants underreported receiving occupational therapy, and one underreported receiving speech and language therapy. Counts presented reflect the chart-verified (corrected) values.

**Supplementary Table 4. Relationship between baseline clinical characteristics and symptom change [follow-up vs. baseline] in the longitudinal functional neurological disorder cohort.**

| **Outcome**  **Variable** | **Baseline**  **Variable** | **Test**  **Statistic** | **Uncorrected**  ***p*-value** | **FDR- corrected**  ***p*-value** |
| --- | --- | --- | --- | --- |
| **SOMS:CD Change** |  |  |  |  |
|  | Age at consent | 0.008^a^ | 0.964 | 1.000 |
|  | Biological sex | 56.500^b^ | 1.000 | 1.000 |
|  | Illness duration | -0.329^a^ | 0.066 | 0.640 |
|  | Interval follow-up period | 0.220^a^ | 0.226 | 0.640 |
|  | Race (white) | 84.500^b^ | 0.106 | 0.640 |
|  | Married | 129.500^b^ | 0.954 | 1.000 |
|  | College graduate | 95.000^b^ | 0.550 | 0.946 |
|  | Employed full-time (or full-time student) | 96.000^b^ | 0.354 | 0.860 |
|  | On medical disability or worker's compensation | 107.500^b^ | 0.934 | 1.000 |
|  | Functional motor disorder | 59.000^b^ | 0.674 | 0.955 |
|  | Functional seizures | 148.000^b^ | 0.123 | 0.640 |
|  | SSRI/SNRI use | 106.000^b^ | 0.509 | 0.946 |
|  | Baseline BDI-II | -0.023^a^ | 0.902 | 1.000 |
|  | Baseline STAI-total | 0.098^a^ | 0.595 | 0.946 |
|  | Baseline PCL-5 | 0.093^a^ | 0.612 | 0.946 |
|  | Baseline CTQ-abuse | -0.237^a^ | 0.191 | 0.640 |
|  | Baseline CTQ-neglect | -0.230^a^ | 0.206 | 0.640 |
| **PHQ-15 Change** |  |  |  |  |
|  | Age at consent | 0.001^c^ | 0.994 | 0.994 |
|  | Biological sex | 0.225^d^ | 0.824 | 0.984 |
|  | Illness duration | 0.004^a^ | 0.982 | 0.994 |
|  | Interval follow-up period | 0.138^a^ | 0.451 | 0.982 |
|  | Race (white) | 0.266^d^ | 0.792 | 0.984 |
|  | Married | 0.451^d^ | 0.655 | 0.984 |
|  | College graduate | -0.168^d^ | 0.868 | 0.984 |
|  | Employed full-time (or full-time student) | 1.155^d^ | 0.257 | 0.945 |
|  | On medical disability or worker's compensation | 66.000^b^ | 0.075 | 0.945 |
|  | Functional motor disorder | 1.105^d^ | 0.278 | 0.945 |
|  | Functional seizures | 81.500^b^ | 0.252 | 0.945 |
|  | SSRI/SNRI use | 128.500^b^ | 0.862 | 0.984 |
|  | Baseline BDI-II | 0.135^a^ | 0.462 | 0.982 |
|  | Baseline STAI-total | 0.035^c^ | 0.851 | 0984 |
|  | Baseline PCL-5 | 0.152^a^ | 0.405 | 0.982 |
|  | Baseline CTQ-abuse | 0.250^a^ | 0.167 | 0.945 |
|  | Baseline CTQ-neglect | 0.101^a^ | 0.583 | 0.984 |
|  | Baseline CTQ-neglect | 106.500^b^ | 0.525 | 1.000 |
| **CGI-I Improved vs. Not Improved** |  |  |  |  |
|  | Age at consent | -0.424^d^ | 0.675 | 1.000 |
|  | Biological sex | 0.647^e^ | 1.000 | 1.000 |
|  | Illness duration | 177.000^b^ | 0.042* | 0.417 |
|  | Interval follow-up period | 137.000^b^ | 0.618 | 1.000 |
|  | Race (white) | 0.444^e^ | 0.629 | 1.000 |
|  | Married | 0.005^f^ | 0.946 | 1.000 |
|  | College graduate | 1.750^e^ | 0.699 | 1.000 |
|  | Employed full-time (or full-time student) | 0.933^e^ | 1.000 | 1.000 |
|  | On medical disability or worker's compensation | 1.038^e^ | 1.000 | 1.000 |
|  | Functional motor disorder | 2.550^e^ | 0.374 | 1.000 |
|  | Functional seizures | 0.161^e^ | 0.049* | 0.417 |
|  | SSRI/SNRI use | 0.042^f^ | 0.837 | 1.000 |
|  | Baseline BDI-II | 127.500^b^ | 0.893 | 1.000 |
|  | Baseline STAI-total | 0.929^d^ | 0.360 | 1.000 |
|  | Baseline PCL-5 | 124.500^b^ | 0.985 | 1.000 |
|  | Baseline CTQ-abuse | 101.500^b^ | 0.409 | 1.000 |
|  | Baseline CTQ-neglect | 106.500^b^ | 0.525 | 1.000 |

*indicates uncorrected p-value <0.05. ^a^Test statistic corresponds to Spearman correlation. ^b^Test statistic corresponds to Mann-Whitney U test. ^c^Test statistic corresponds to Pearson correlation. ^d^Test statistic corresponds to T-test. ^e^Test statistic corresponds to Fisher’s exact test. ^f^Test statistic corresponds to chi-squared test. CGI-I improved includes “much improved” and “improved”, while CGI-I not-improved includes “unchanged” and “worse,” based on patient-reported Clinical Global Impression of Improvement (CGI-I) scale scores. False discovery rate (FDR) correction was performed across all baseline variables for a given symptom outcome measure. SOMS:CD, Screening for Somatoform Symptoms-7 Subscale for Conversion Disorder; PHQ-15, Patient Health Questionnaire-15; BDI-II, Beck Depression Inventory-II; STAI, Spielberg State-Trait Anxiety Inventory; PCL-5, PTSD Checklist 5; CTQ, Childhood Trauma Questionnaire.

**SUPPLEMENTARY FIGURES**

**Supplementary Fig. 1.** Upset plot of functional neurological symptoms across the functional neurological disorder (FND) cohort. Vertical bars reflect the number of individuals with each combination of co-occurring symptoms, as shown by multiple darkened circles connected by lines, or single symptom presentation indicated by one darkened circle. Horizontal bars reflect the total number of individuals who experienced each individual symptom (with or without other co-occurring symptoms).

**Supplementary Fig. 2.** Cerebellar findings associated with functional neurological disorder (FND) symptom change. (A) Baseline cerebellar weighted-degree values positively associated with FND symptom change (SOMS:CD change scores; N=32). Specifically, within the cerebellum, findings were localized to the left hemisphere, spanning cerebellar lobules VI, Crus I, Crus II, VIIb, VIIIa, VIIIb, and X, based on the spatially unbiased atlas template of the cerebellum and brainstem (SUIT atlas^1^). (B) Longitudinal changes in cerebellar weighted-degree values associated with FND symptom change (SOMS:CD change scores; N=28). Specifically, within the cerebellum, findings were localized to the right hemisphere, spanning cerebellar lobules VI, Crus 1, Crus II, VIIb, VIIIa, and VIIIb, based on the SUIT atlas^1^. Colors in the maps reflect the z-statistic for the primary general linear model adjusting for age, sex, SSRI/SNRI use, mean framewise displacement, elapsed time between baseline and follow-up sessions, and baseline SOMS:CD score (z-statistic>1.96; p<0.05 cluster-corrected for multiple comparisons). White outlines reflect regions that also held across post-hoc corrections for FND phenotype (FND-seizure yes/no). Images are shown in neurological view (left = left). SOMS:CD, SOMS:CD, Screening for Somatoform Symptoms-7 Subscale for Conversion Disorder; SSRI/SNRI, selective serotonin reuptake inhibitor/serotonin norepinephrine reuptake inhibitor.

**Supplementary Fig. 3.** Results from analyses relating functional neurological disorder (FND) symptom change scores to baseline resting-state functional connectivity metrics (*left,* whole-brain weighted-degree; *middle,* cortical integration; *right*, cortical segregation; N=32). The top row depicts results from the primary adjustment for age, sex, SSRI/SNRI use, mean framewise displacement (FD), elapsed time between baseline and follow-up sessions, and baseline SOMS:CD score; the second row depicts results from the *post-hoc* adjustment controlling additionally for FND phenotype (FND-seizure yes/no); the third row depicts results from the *post-hoc* adjustment controlling additionally for baseline BDI-II, STAI-total, and PCL-5 scores; the bottom row depicts results from the *post-hoc* adjustment controlling additionally for CTQ-abuse and CTQ-neglect scores. Colors reflect the z-statistic for the corresponding general linear (z-statistic>1.96; p<0.05 cluster-corrected for multiple comparisons). Volumetric brain maps are visualized on the surface for display purposes only; subcortical results in volume are shown with the left hemisphere on the left side of the image. SOMS:CD, Screening for Somatoform Symptoms-7 Subscale for Conversion Disorder; SSRI/SNRI, selective serotonin/norepinephrine reuptake inhibitor; FD, framewise displacement; BDI-II, Beck Depression Inventory-II; STAI-total, State Trait Anxiety Inventory-Total; PCL-5, PTSD Checklist-5; CTQ, Childhood Trauma Questionnaire.

**Supplementary Fig. 4.** Results from analyses relating functional neurological disorder (FND) symptom change scores to baseline cortical integration (left; N=32) and longitudinal changes in cortical integration (right; N=28) between follow-up and baseline across *post-hoc* adjustments for CTQ domains. Exploratory *post-hoc* analyses were separately conducted for the five trauma subdomains of the CTQ: physical abuse, emotional abuse, sexual abuse, physical neglect, and emotional neglect. The top row depicts results from the primary adjustment for age, sex, SSRI/SNRI use, mean framewise displacement (FD), elapsed time between baseline and follow-up sessions, and baseline SOMS:CD score; the second row depicts results from the *post-hoc* adjustment controlling additionally for CTQ Emotional Abuse; the third row depicts results from the *post-hoc* adjustment controlling additionally for CTQ Physical Abuse; the fourth row depicts results from the *post-hoc* adjustment controlling additionally for CTQ Sexual Abuse; the fifth row depicts results from the *post-hoc* adjustment controlling additionally for CTQ Emotional Neglect; the bottom row depicts results from the *post-hoc* adjustment controlling additionally for CTQ Physical Neglect. Colors reflect the z-statistic for the corresponding general linear (z-statistic>1.96; p<0.05 cluster-corrected for multiple comparisons). Volumetric brain maps are visualized on the surface for display purposes only; subcortical results in volume are shown with the left hemisphere on the left side of the image. SOMS:CD, Screening for Somatoform Symptoms-7 Subscale for Conversion Disorder; SSRI/SNRI, selective serotonin/norepinephrine reuptake inhibitor; FD, framewise displacement; CTQ, Childhood Trauma Questionnaire.

**Supplementary Fig. 5.** Network connectivity of baseline cortical integration predictors associated with functional neurological disorder (FND) symptom change (N=32). Only three networks containing the most significant voxels are displayed: (**A**) ventral attention network; (**B**) frontoparietal network; (**C**) default mode network (DMN). For each of these networks, we extracted the average time series of all significant voxels for use in seed-to-voxel connectivity analyses, to then characterize the networks they were functionally integrating across. Pie charts detail the percent of significant connections from the seed network to each of the other Yeo et al. (2011) networks^2^. Self-connections (e.g., DMN-to-DMN) were excluded, as cortical integration analyses focused on cross-network connectivity. Charts excluded networks with fewer than 1.0% of connections.

**Supplementary Fig. 6.** Results from analyses relating physical symptom change scores to baseline resting-state functional connectivity metrics (*left,* whole-brain weighted-degree; *middle,* cortical integration; *right*, cortical segregation; N=32). The top row depicts results from the primary adjustment for age, sex, SSRI/SNRI use, mean framewise displacement (FD), elapsed time between baseline and follow-up sessions, and baseline PHQ-15 score; the second row depicts results from the *post-hoc* adjustment controlling additionally for FND phenotype (FND-seizure yes/no); the third row depicts results from the *post-hoc* adjustment controlling additionally for baseline BDI-II, STAI-total, and PCL-5 scores; the bottom row depicts results from the *post-hoc* adjustment controlling additionally for CTQ-abuse and CTQ-neglect scores. Colors reflect the z-statistic for the corresponding general linear (z-statistic>1.96; p<0.05 cluster-corrected for multiple comparisons). Volumetric brain maps are visualized on the surface for display purposes only. PHQ-15, Patient Health Questionnaire-15; SSRI/SNRI, selective serotonin/norepinephrine reuptake inhibitor; FD, framewise displacement; BDI-II, Beck Depression Inventory-II; STAI-total, State Trait Anxiety Inventory-Total; PCL-5, PTSD Checklist-5; CTQ, Childhood Trauma Questionnaire.

**Supplementary Fig. 7.** Results from analyses relating FND symptom change scores to changes in resting-state functional connectivity metrics (*left,* whole-brain weighted-degree; *middle,* cortical integration; *right*, cortical segregation; N=28) between follow-up and baseline. The top row depicts results from the primary adjustment for age, sex, SSRI/SNRI use, mean framewise displacement (FD), elapsed time between baseline and follow-up sessions, and baseline SOMS:CD score; the second row depicts results from the *post-hoc* adjustment controlling additionally for FND phenotype (FND-seizure yes/no); the third row depicts results from the *post-hoc* adjustment controlling additionally for baseline BDI-II, STAI-total, and PCL-5 scores; the fourth row depicts results from the *post-hoc* adjustment controlling additionally for CTQ-abuse and CTQ-neglect scores; the fifth row depicts results from the *post-hoc* adjustment controlling additionally for a change in BDI-II scores between baseline and follow-up; the bottom row depicts results from the *post-hoc* adjustment controlling additionally for a change in STAI-total scores between baseline and follow-up. Note: while we focus on whether or not findings in the primary analyses hold for various adjustments, there are instances where the additional *post-hoc* adjustments led to the identification of new results, such as the adjustment for BDI-II, STAI-total, and PCL-5 for cortical integration and segregation; this underscores the continued need to understand convergent and disorder-specific pathways in the neurocircuitry of FND. Colors reflect the z-statistic for the corresponding general linear (z-statistic>1.96; p<0.05 cluster-corrected for multiple comparisons). Volumetric brain maps are visualized on the surface for display purposes only; subcortical results in volume are shown with the left hemisphere on the left side of the image. SOMS:CD, Screening for Somatoform Symptoms-7 Subscale for Conversion Disorder; SSRI/SNRI, selective serotonin/norepinephrine reuptake inhibitor; FD, framewise displacement; BDI-II, Beck Depression Inventory-II; STAI-total, State Trait Anxiety Inventory-Total; PCL-5, PTSD Checklist-5; CTQ, Childhood Trauma Questionnaire.

**Supplementary Fig. 8.** Longitudinal changes in cortical integration functional connectivity associated with physical symptom change (Δ PHQ-15 scores) between baseline and follow-up (N=28). (**A**) Regions with changes in cortical integration that were significantly positively associated with physical symptom changes. Specifically, symptom improvement was associated with increased integration at follow-up compared to baseline in the left lateral occipital pole and occipital fusiform gyrus, while symptom worsening was associated with decreased integration in these regions. Colors in the map reflect the z-statistic for the primary general linear model adjusting for age, sex, SSRI/SNRI use, mean framewise displacement, elapsed time between baseline and follow-up sessions, and baseline PHQ-15 score (z-statistic>1.96; p<0.05 cluster-corrected for multiple comparisons). Statistical maps for additional post-hoc corrections are visualized in **Supplementary Fig. 9**. (**B**). Scatterplot of PHQ-15 change scores on the x-axis and mean cortical integration change values averaged across significant voxels on the y-axis. Datapoints in the scatterplot reflect the mean change in integration value and corresponding PHQ-15 change score for each individual participant. The shaded region reflects the 95% confidence interval. For visualization purposes, change scores are plotted with negative values on the right to reflect symptom improvement. (**C**) Bar chart summarizing between-network connectivity increases using all voxels in panel (A) as a visual network seed. Only networks with increased connections to the visual network seed at follow-up compared to baseline are shown, given that an increase in integration for these voxels was associated with symptom improvement. Connections with the frontoparietal, limbic, and default mode networks were higher at follow-up compared to baseline. F/U, follow-up; PHQ-15, Patient Health Questionnaire-15; VIS, visual network; FPN, frontoparietal network; LIM, limbic network; DMN, default mode network.

**Supplementary Fig. 9.** Results from analyses relating physical symptom change scores to changes in resting-state functional connectivity metrics (*left,* whole-brain weighted-degree; *middle,* cortical integration; *right*, cortical segregation; N=28) between follow-up and baseline. The top row depicts results from the primary adjustment for age, sex, SSRI/SNRI use, mean framewise displacement (FD), elapsed time between baseline and follow-up sessions, and baseline PHQ-15 score; the second row depicts results from the *post-hoc* adjustment controlling additionally for FND phenotype (FND-seizure yes/no); the third row depicts results from the *post-hoc* adjustment controlling additionally for BDI-II, STAI-total, and PCL-5 scores; the fourth row depicts results from the *post-hoc* adjustment controlling additionally for CTQ-abuse and CTQ-neglect scores; the fifth row depicts results from the *post-hoc* adjustment controlling additionally for a change in BDI-II scores between baseline and follow-up; the bottom row depicts results from the *post-hoc* adjustment controlling additionally for a change in STAI-total scores between baseline and follow-up. Colors reflect the z-statistic for the corresponding general linear (z-statistic>1.96; p<0.05 cluster-corrected for multiple comparisons). Volumetric brain maps are visualized on the surface for display purposes only. PHQ-15, Patient Health Questionnaire-15; SSRI/SNRI, selective serotonin/norepinephrine reuptake inhibitor; FD, framewise displacement; BDI-II, Beck Depression Inventory-II; STAI-total, State Trait Anxiety Inventory-Total; PCL-5, PTSD Checklist-5; CTQ, Childhood Trauma Questionnaire.

**Supplementary Fig. 10.** Contextualization of longitudinal changes in cortical integration-level network connectivity in comparison to healthy controls. Boxplots depict mean cortical integration values for overlapping voxels identified across baseline predictors and longitudinal change analyses (depicted in **Figure 7**), stratified by (A) BDI-II symptom change and (B) STAI-total symptom change. Plots are shown for the top 20% of FND participants with the greatest BDI-II or STAI-total symptom improvement (N=5; baseline: dark green, follow-up: light green), the bottom 20% of participants with the greatest BDI-II or STAI-total symptom worsening (N=5; baseline: dark blue, follow-up: light blue), and healthy control participants (N=50; orange). Individual datapoints represent the mean cortical integration value across overlapping voxels for a single participant at the indicated timepoint. For both BDI-II and STAI-total, participants with the greatest improvement showed a pattern of lower baseline cortical integration and higher follow-up cortical integration. This is in contrast to our analyses stratifying by SOMS:CD change scores (depicted in **Figure 7**), where those who improved the most showed significantly elevated baseline cortical integration that decreased at follow-up, consistent with the idea of hyperconnectivity normalizing as FND symptoms improved. These findings suggest that the right anterior insula integration result may be more specifically related to core FND symptom change than to other symptom trajectories. BDI-II, Beck Depression Inventory-II, STAI-total, State-Trait Anxiety Inventory-Total Score; SOMS:CD, Screening for Somatoform Symptoms-7 Subscale for Conversion Disorder.

**Supplementary References**

1.	Diedrichsen J, Balsters JH, Flavell J, Cussans E, Ramnani N. A probabilistic MR atlas of the human cerebellum. *NeuroImage*. 2009;46(1):39-46. doi:10.1016/j.neuroimage.2009.01.045

2.	Yeo BTT, Krienen FM, Sepulcre J, et al. The organization of the human cerebral cortex estimated by intrinsic functional connectivity. *Journal of Neurophysiology*. 2011;106(3):1125. doi:10.1152/jn.00338.2011
